# Therapeutic potential of traditional Chinese medicine and mechanisms for the treatment of type 2 diabetes mellitus

**DOI:** 10.1186/s13020-025-01222-x

**Published:** 2025-10-04

**Authors:** Miao Wang, Qing Yang, Ye Li, Yang Zhao, Junbo Zou, Fei Luan, Xiujuan Peng, Zhuangzhuang Huang, Feng Liu

**Affiliations:** 1 School of Pharmacy, Shaanxi University of Chinese Medicine, Xianyang, 712046 People’s Republic of China; 2 Key Laboratory of Chemical Substances and Biological Effects of Traditional Chinese Medicine, Shaanxi Institute of International Trade & Commerce, Xi’an, 712046 People’s Republic of China; 3 Scientific Research Department, Shaanxi Buchang Pharmaceutical Co. Ltd, Xi’an, 710075 People’s Republic of China

**Keywords:** Type 2 diabetes mellitus, Pathological mechanism, Traditional Chinese medicine, Chinese herbal formula, Active ingredients

## Abstract

Diabetes, a chronic metabolic non-communicable disease, has become prevalent worldwide. Its prevalence is increasing at an alarming rate, and the affected population is becoming increasingly younger, posing a serious threat to both individual health and social well-being. Notably, type 2 diabetes mellitus (T2DM) accounts for more than 90% of all diabetes cases, a statistic that underscores its dominant position in the disease burden. Despite substantial progress in the field of diabetes treatment, existing diabetes medications still fail to fully address the complex and pressing clinical needs of patients. Therefore, the development of new therapeutic drugs for T2DM is of crucial significance. Traditional Chinese medicine (TCM) has shown significant potential in alleviating the symptoms of T2DM and improving patients' quality of life, providing an effective complementary approach to conventional treatments. This study systematically clarifies and reviews the pathogenesis of T2DM from multiple dimensions, including inflammation, oxidative stress, ferroptosis, pyroptosis, necroptosis, autophagy, and gut microbiota. It also examines and summarizes TCM compound formulations and their main active ingredients that can improve the disease, and elucidates the disease-improving effects of these TCM ingredients targeting the aforementioned mechanisms. Studies have shown that TCM plays an important role in improving T2DM by targeting key mechanisms such as insulin resistance, β-cell damage, and glucose-lipid metabolism disorders. This not only provides valuable ideas and reference basis for researchers in endocrine basic research, pharmacologists, and clinicians to develop TCM with low toxicity and side effects but also opens up new research directions for the treatment of T2DM and lays a theoretical foundation for new drug development.

**Graphical Abstract:**

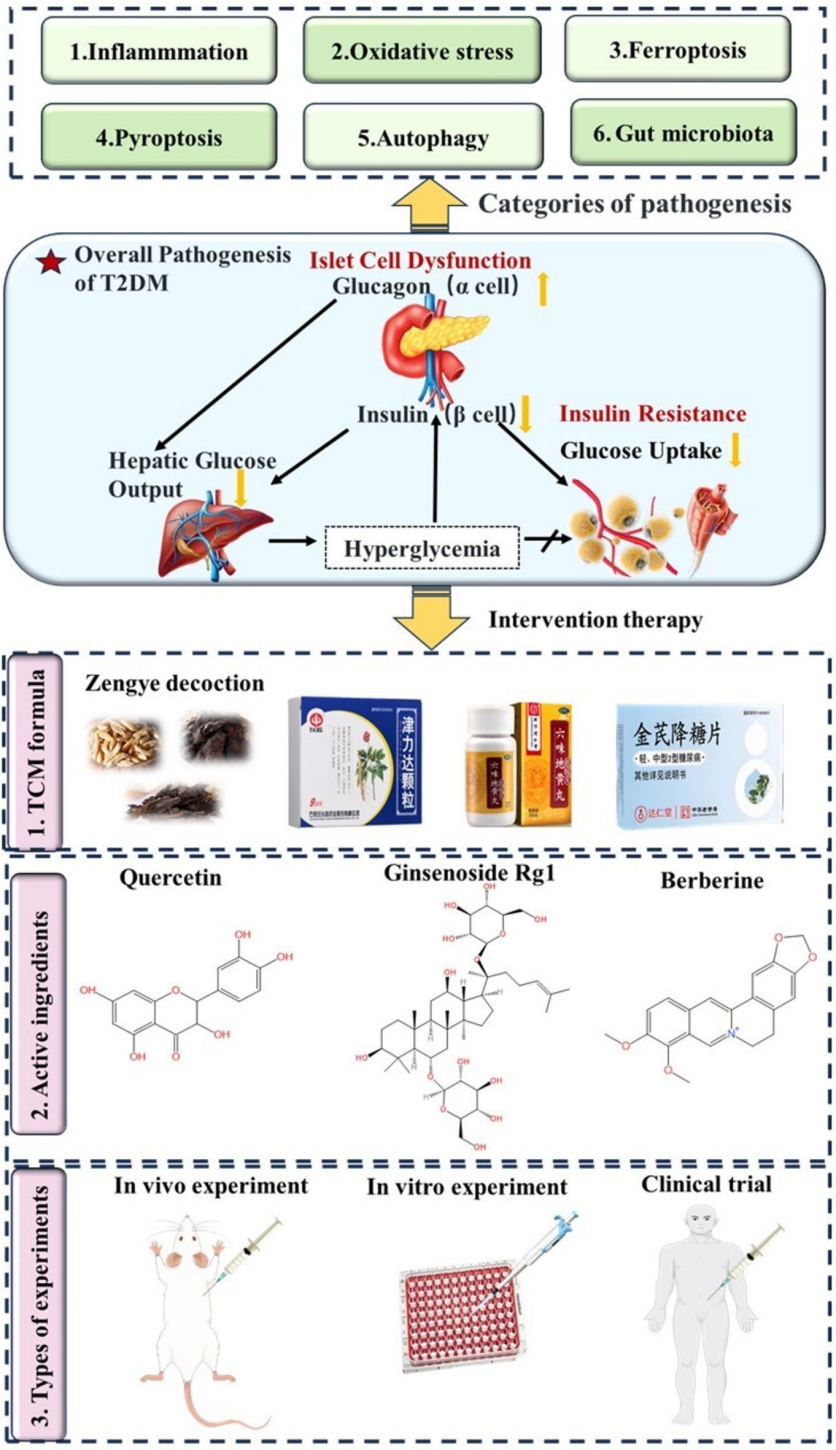

## Highlights

- Type 2 diabetes mellitus remains a leading cause of death worldwide.
- Herbal medicine as a potential treatment for type 2 diabetes mellitus.
- Herbal medicine has a synergistic effect with multiple components, targets, and pathways, and fewer side effects.
- Conduct a deep assessment of herbal medicine research status.

## Introduction

Diabetes mellitus is a group of metabolic disorders marked by persistent high blood glucose. It's a leading global cause of death, with its incidence rising [1, 2]. In 2019, around 463 million adults worldwide had diabetes, and the prevalence is increasing alarmingly, expected to reach 578 million by 2030 and 700 million by 2045 [3]. It has four subtypes: type 1, type 2, gestational, and others [4]. Type 2 diabetes mellitus (T2DM), formerly known as adult-onset diabetes, accounts for more than 90% of diabetes cases in Western populations [5]. T2DM arises from insulin resistance (IR) and β-cell dysfunction, leading to impaired glucose uptake despite compensatory hyperinsulinemia, followed by progressive insulin deficiency [6]. This is accompanied by complications like nephropathy, neuropathy, and retinopathy, which severely affect patients' quality of life and increase the medical burden. Thus, it is crucial to find new treatments to alleviate the condition of T2DM.

In recent years, in addition to traditional antidiabetic drugs (such as biguanides, sulfonylureas, meglitinides, and insulin), new classes of drugs have emerged, including DPP-4 inhibitors, GLP-1 receptor agonists, SGLT-2 inhibitors, and GLP-1/GIP dual receptor agonists [7]. These drugs can stimulate pancreatic β cell insulin secretion, boost insulin sensitivity, lower blood glucose, and enhance insulin release via GLP-1 or GLP-1/GIP agonists, helping patients relieve symptoms [8]. However, they come with notable side effects such as hypoglycaemia, gastrointestinal issues, and higher infection risk, causing patient dependency [9]. Moreover, the development of highly effective hypoglycemic drugs is encumbered by the twin challenges of exorbitant research and development costs and stringent patent restrictions. These factors not only keep the prices of such medications at prohibitively high levels but also significantly exacerbate the difficulty for patients to access them. Thus, addressing this issue has become a task of great urgency and critical importance.

Traditional Chinese Medicine (TCM) plays a broad and profound role in the prevention and treatment of diseases [10]. China has carried out many experiments showing TCM's effectiveness in improving T2DM [11, 12]. T2DM is classified as "diabetes" in TCM, first noted as "Xiao ke" in the "Yellow Emperor’s Inner Canon" (200 BC), caused by factors like genetic weakness, obesity, kidney deficiency, diet, stress, and yin—yang imbalance. The core pathogenesis is yin deficiency with dry heat, mainly affecting the kidneys and also involving other organs, resulting in qi deficiency, blood stasis, etc. Current T2DM treatment strategies in TCM focus on heat-clearing and yin-nourishing principles, with reported efficacy of classical formulations including Zengye Decoction [13], Gegen Qinlian Decoction [14] and Liuwei Dihuang Pill [15]. Furthermore, after conducting in-depth research on the major bioactive components in TCM, pharmacological researchers have found that berberine [16], quercetin [17], and puerarin [18] and other components provide supplementary support for disease treatment through pathways such as improving insulin sensitivity, regulating inflammatory responses, and modulating metabolic disorders. While inflammatory and oxidative stress mechanisms in T2DM are relatively well-established, emerging mechanisms such as ferroptosis, pyroptosis, autophagy, and gut microbiota remain insufficiently studied. The multi-component, multi-target nature of TCM presents unique challenges in elucidating its therapeutic mechanisms. This review synthesizes current research progress, evaluates therapeutic potential and clinical applications, systematically analyses the efficacy, safety, and challenges of TCM in treating T2DM, while proposing potential solutions and outlining future research directions for TCM in diabetes management.

## Materials and methods

**Fig. 1:**
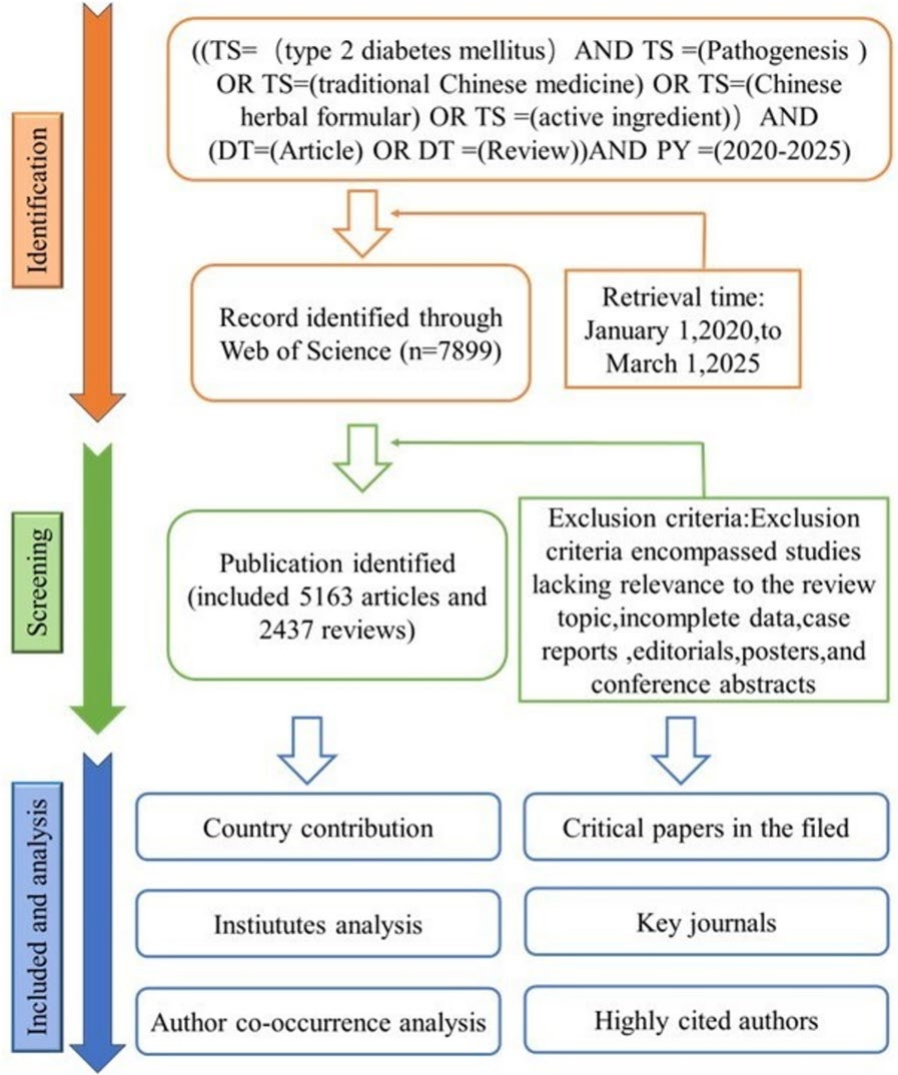
Flowchart of screening process

In this study, the keywords "Type 2 Diabetes", "pathogenesis", "inflammation", "oxidative stress","ferroptosis", "pyroptosis", "autophagy", "gut microbiota", "herbal formulas" and "active ingredients", or "pathogenesis" and "herbal formulas" or "active ingredients" interactions were used in PubMed (https:pubmed.ncbi.nlm.nih.gov), Science Network (http://apps.webofknowledge.com/), China National Knowledge Infrastructure (http://www.cnki.net), and Baidu Academic (https://xueshu.baidu.com/), and the Wanfang database (https:www.wanfangdata.com.cn) for database retrieval. We focused on global literature published in the past five years, up to March 2025 and followed PRISMA 2020 guidelines. The published Chinese and English literature in this review is directly related to the pathogenesis of T2DM caused by IR and damage to the structure and function of islet β-cells, resulting in disorders of glucose and lipid metabolism, and the mechanism of action of TCM compound preparations and active ingredients in the treatment of T2DM. All published studies in both Chinese and English, including in vitro, in vivo experiments, and clinical trials, were included. Initially, articles were selected based on their titles and abstracts. Finally, a detailed analysis of the full texts was conducted, covering aspects such as sources, access pathways, herbal components, mechanisms of action, toxicology, side effects and bioavailability. Studies involving T2DM complications, as well as incomplete data, case reports, editorials, posters, and conference abstracts, were excluded (Fig. 1). This review follows the guidelines outlined in the Preferred Reporting Items for Systematic Reviews and Meta-Analysis (PRISMA) Statement [19].

## Pathological mechanism of T2DM

**Fig. 2:**
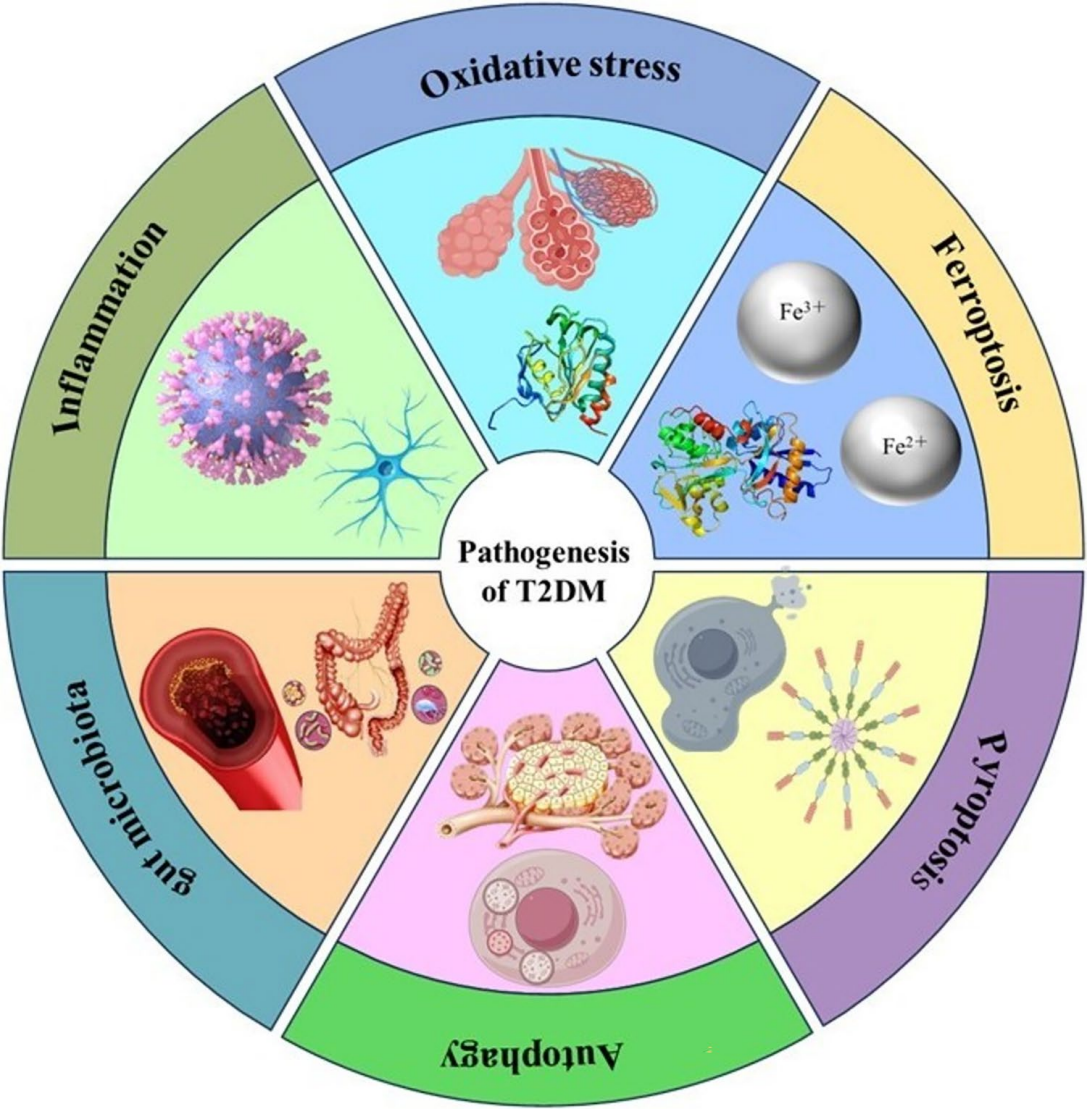
The seven major mechanisms influencing the pathogenesis of T2DM. Such as inflammation, oxidative stress, ferroptosis, pyroptosis, autophagy, and gut microbiota

The pathogenesis of T2DM is intricate, with IR as a key pathological feature [20]. IR means a reduced response of insulin—targeted tissues to normal insulin levels, mainly due to post-insulin receptor (InsR) signal transduction problems [6]. InsR function defects directly lead to IR [21]. The InsR, a tyrosine kinase receptor on insulin target cell membranes, binds insulin, activates intracellular receptors through phosphorylation, transduces insulin signals, changes target cell physiology, and helps glucose enter cells [22]. Meanwhile, glucose metabolism enzymes regulate glucose synthesis and metabolism in target cells to keep blood glucose balanced. InsR malfunction impairs insulin secretion and target cell activation, lowering insulin sensitivity. Continuous hyperglycemia stops cells from taking up glucose, raising blood glucose and causing T2DM [23, 24]. Thus, T2DM pathogenesis is mainly explained from aspects like inflammation, oxidative stress, ferroptosis, pyroptosis, autophagy, and gut microbiota, as shown in Fig. 2.

### Inflammation

**Fig. 3:**
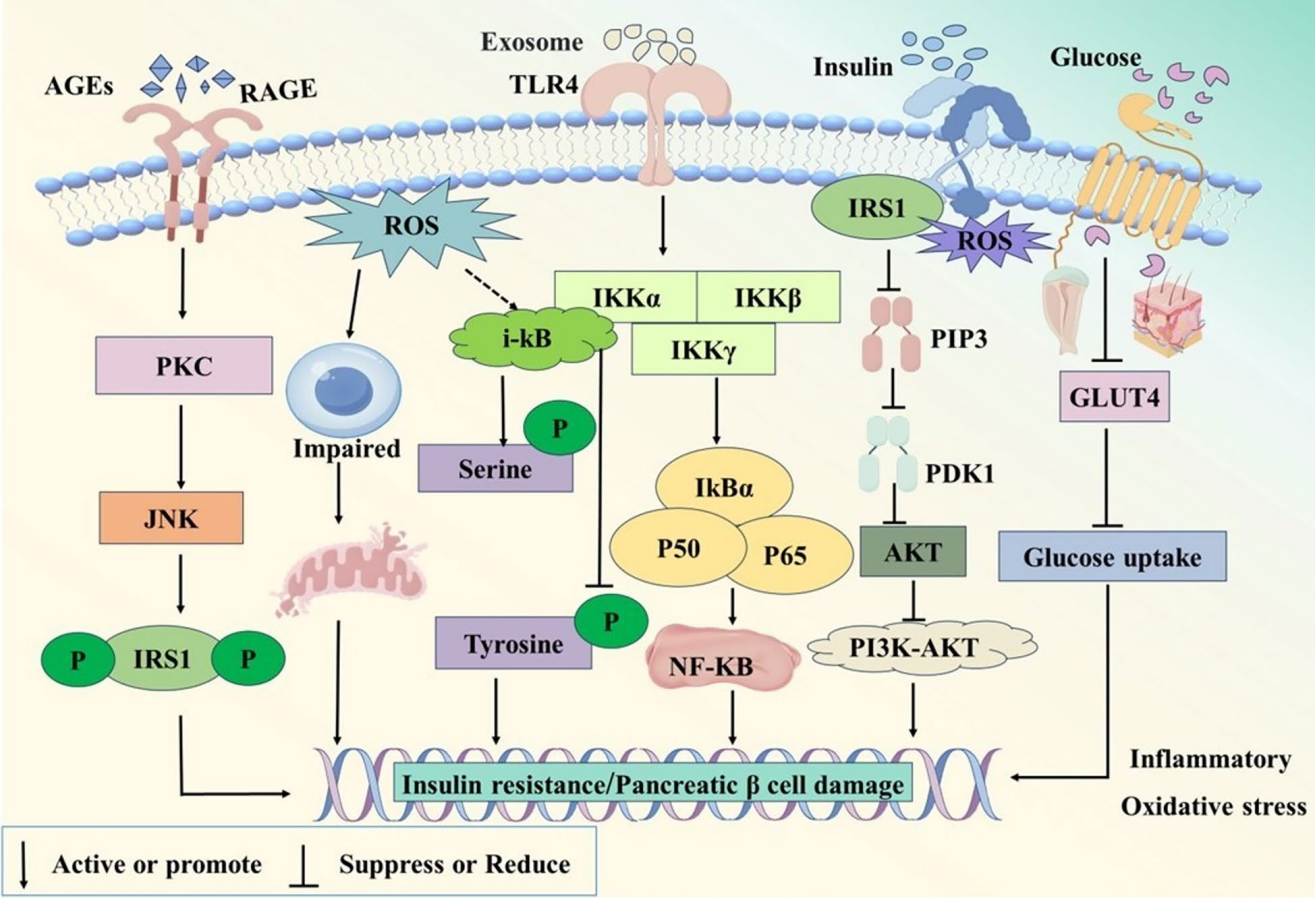
Mechanisms of inflammation and oxidative stress in T2DM. Prolonged hyperglycemia leads to the formation of AGEs through a process of non-enzymatic glycation. These AGEs bind to their primary cellular receptor RAGE, activating downstream signaling pathways such as JNK, which induces oxidative stress and triggers a series of inflammatory responses. Activation of the AGE/RAGE axis results in increased serine phosphorylation and degradation of insulin receptor substrates, thereby obstructing insulin signaling pathways and ultimately leading to IR. Exosomes released from adipose tissue enter cells in a manner dependent on Toll-like receptor 4, inducing phosphorylation of IkBα and translocation of P65/P50 into the nucleus. IRS1 activate AKT through PIP3, PDK1, and other pathways, affecting the PI3K-AKT pathway. Both mechanisms ultimately promote the secretion of inflammatory factors and exacerbate IR. OS interferes with the phosphorylation of IRS1 through various pathways, hindering the transmission of insulin signals. IKK activates the inhibitory subunit IκB, which in turn promotes the activation of NF-κB. Under the stimulation of ROS, there is an increase in serine phosphorylation of insulin receptor substrates, while tyrosine phosphorylation is suppressed, further obstructing insulin signal transduction. ROS can also directly damage pancreatic β-cells, disrupt their mitochondrial structure, and promote apoptosis. Additionally, ROS activates the NF-κB signaling pathway, triggering inflammatory responses in β-cells and altering mitochondrial energy metabolism, which reduces insulin synthesis and secretion, thereby exacerbating the pathogenesis of T2DM. Furthermore, GLUT4 is specifically expressed in insulin-sensitive tissues (such as adipose tissue and skeletal muscle), and OS downregulates GLUT4 expression, leading to decreased glucose uptake and the development of IR

T2DM pathogenesis is closely linked to inflammation (Fig. 3). Pro-inflammatory immune cell infiltration in pancreatic islets and insulin-sensitive tissues (adipose, liver, muscle) drives local inflammation, impairing insulin secretion/sensitivity to induce T2DM. Following the onset of the disease, hyperglycemia and metabolic disorders further activate inflammatory pathways, worsening inflammation and creating a vicious cycle.

#### Damage to pancreatic islet β cells

Under chronic inflammatory conditions, the body persistently secretes a range of pro-inflammatory cytokines (e.g., IL-1β, TNF-α), which specifically target pancreatic islet β cells. These cytokines induce β cell apoptosis and concomitantly suppress insulin synthesis and secretion via the activation of key signaling pathways such as NF-κB [25]. They also directly inhibit insulin gene expression and proinsulin processing, reducing insulin synthesis and secretion at the source. In addition, long-term hyperglycemia (glucotoxicity) and hyperlipidemia (lipotoxicity) synergistically amplify inflammatory responses, accelerate the functional failure of β cells, and ultimately lead to the inability of insulin secretion to compensate for IR, thereby triggering T2DM [26].

#### Induction of insulin resistance

Obesity, a key risk factor for T2DM, elicits adipose tissue dysfunction and localized chronic inflammation. Specifically, adipocytes that undergo excessive proliferation or hypertrophy secrete substantial amounts of free fatty acids (FFA) and pro-inflammatory cytokines (e.g., TNF-α, IL-6, IL-1β), which recruit immune cells to infiltrate adipose tissue. This process drives a phenotypic shift from an M2-polarized "anti-inflammatory" state to an M1-polarized "pro-inflammatory" state, thereby exacerbating inflammatory responses [27]. Ultimately, inflammatory signals reduce insulin sensitivity in liver, muscle and adipose tissue by inhibiting Insulin receptor (IRS) phosphorylation and activating stress pathways like JNK/p38. Thomou et al. [28] reported that insulin-secreting β cells and insulin-sensitive organs communicate with immune cells or endothelial cells via exosomes, thereby regulating glucose homeostasis and IR. In line with this regulatory mechanism, when exosomes released by adipose tissue enter target cells through TLR4, they activate the IKK complex, which induces phosphorylation and degradation of IkBα. The released NF-κB p65/p50 heterodimer then translocates into the nucleus, promoting the expression of pro-inflammatory factors such as TNF-α and IL-6. These factors in turn disrupt the insulin signaling pathway, ultimately leading to IR [29, 30]. Chronic hyperglycemia enhances the generation of advanced glycation end products (AGEs). Upon binding to their cognate receptor RAGE, AGEs trigger the activation of protein kinase C (PKC) through specific adaptor proteins, which subsequently activates signaling cascades including JNK, MAPK/ERK, TGF-β, and NF-κB. These pathways disrupt insulin signaling via mechanisms such as promoting serine phosphorylation of IRS, ultimately resulting in IR, hyperglycemia, and exacerbation of T2DM [31–33].

### Oxidative stress

Oxidative stress (OS), is an imbalance between the body's oxidation and antioxidant defense systems. Reactive oxygen species (ROS) from OS are usually neutralized by cellular antioxidant mechanisms. When the damaging effects of ROS exceed the compensatory capacity of the antioxidant system, oxidative damage occurs [34]. The specific pathogenesis is illustrated in Fig. 3.

#### The effect on islet β cells

Pancreatic β-cells synthesize and secrete insulin, and their functional decline, leading to insulin deficiency, is a key factor in T2DM development. OS significantly contributes to β-cell dysfunction. ROS indirectly impair β-cell function by disrupting insulin signaling, activating NF-κB signaling to trigger inflammation, inhibiting pancreatic and duodenal homeobox 1 nuclear-cytoplasmic translocation, and suppressing mitochondrial energy metabolism. These effects reduce insulin synthesis and secretion, accelerating T2DM progression [35–37].

##### The effect on insulin resistance

In T2DM, IR manifests as a decrease in insulin sensitivity in target tissues of insulin action, such as the liver, muscle, and fat, which leads to disorders of glucose and lipid metabolism in the body. The essential cause of impaired insulin sensitivity is a disorder in insulin signal transduction. The InsR and IRS are key components of this pathway, with InsR serving as the initial signal transducer and IRS acting as a bridge to downstream elements. OS disrupts the phosphorylation of InsR and IRS through various pathways, impeding insulin signaling. IKK activates the inhibitory subunit IκB of NF-κB. In the presence of ROS, IKK serves as a serine/threonine kinase for InsR and IRS, promoting serine phosphorylation while inhibiting normal tyrosine phosphorylation, thereby hindering insulin signal transductionm [38]. The role of ROS is to disrupt the recruitment of 3-Phosphoinositide-Dependent Protein Kinase 1(PDK1) by Phosphatidylinositol-3,4,5-trisphosphate (PIP3) and inhibit the activation of AKT by PDK1. This prevents the normal transmission of insulin signals through the PI3K-AKT pathway, ultimately leading to IR [39]. Additionally, Glucose Transporter 4 (GLUT4), an important member of the GLUT family, is specifically expressed in insulin sensitive tissues such as adipose tissue, and myocardium, and affects insulin signalling. Ma et al. [40] showed that insulin helps GLUT4 move from intracellular compartments to the plasma membranes of adipocytes and muscle cells, acutely boosting glucose uptake. Notably, reduced GLUT4 expression weakens glucose uptake ability and contributes to IR development [41].

### Ferroptosis

**Fig. 4:**
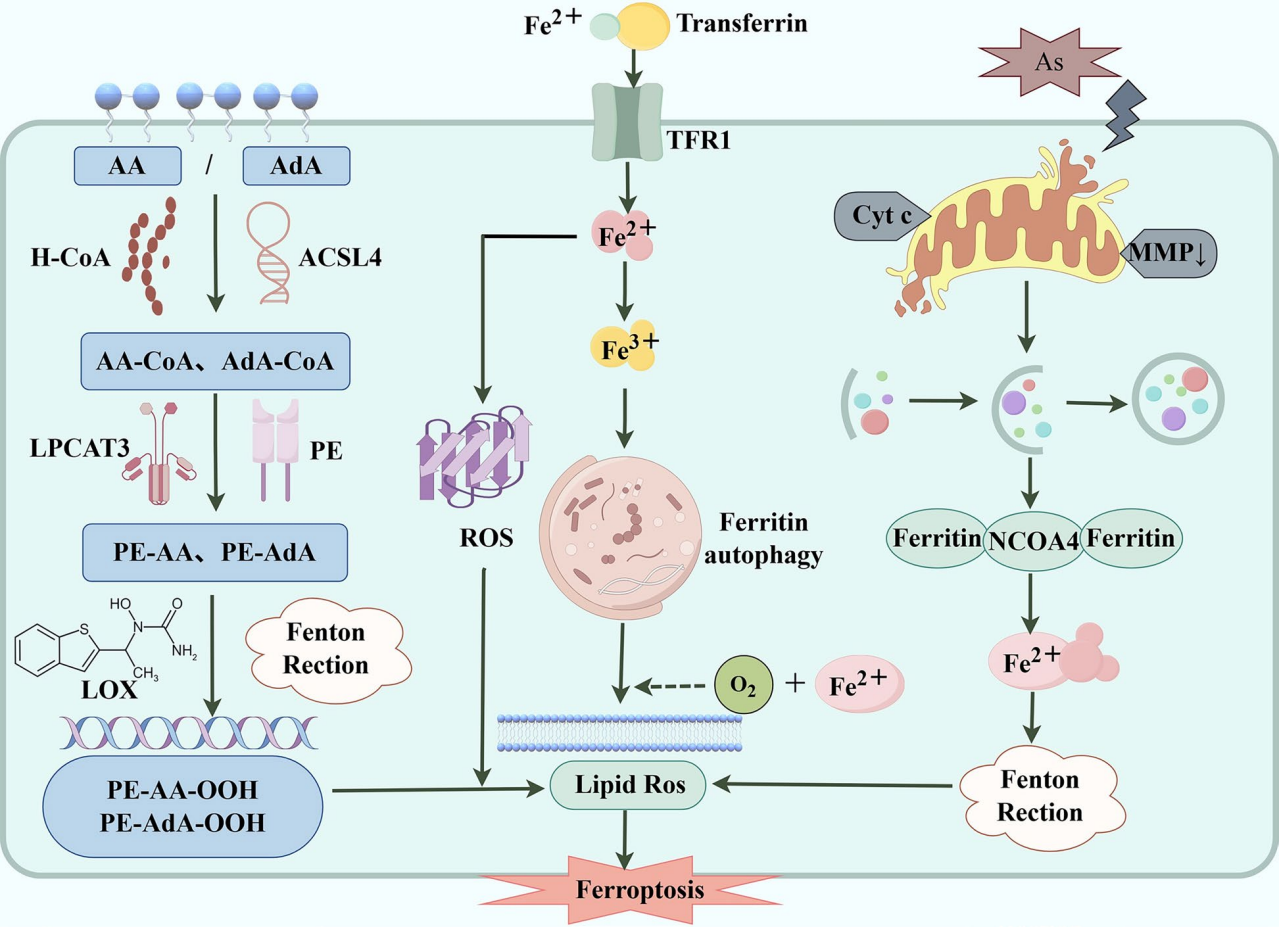
The pathogenic mechanisms of ferroptosis in T2DM. ACSL4 activates AA and AdA, participating in fatty acid oxidation and lipid biosynthesis. PE and LPCAT3 further convert the acylated AA and AdA into phosphatidylethanolamine. Under the influence of lipoxygenases and Fenton reactions, this process generates complex peroxides; Exposure to AS reduces the expression of antioxidant enzymes, leading to mitochondrial damage and excess production of mitochondrial MtROS. This situation increases intracellular levels of free iron and the occurrence of iron-dependent autophagy. Meanwhile, coactivator of nuclear receptors 4 promotes the release of more Fe^2^⁺, accelerating the occurrence of the Fenton reaction; Fe^2^⁺ binds to transferrin and enters the cell, promoting the generation of lipid-derived ROS, which subsequently leads to ferroptosis. In the body, Fe^2^⁺ is converted to Fe^3^⁺, inducing the occurrence of ferroautophagy. All of the above ultimately leads to lipid peroxidation and ferroptosis, which in turn triggers the onset of T2DM

Ferroptosis, first proposed by Professor Brent R. Stockwell in 2012, is an iron-dependent non-apoptotic cell death. It's marked by intracellular iron overload and lipid peroxide accumulation [42]. Morphologically, it shows mitochondrial shrinkage, higher membrane density, and reduced or absent cristae [43]. Regulated by pathways related to GPX4, iron, and lipid metabolism. ferroptosis has been linked to T2DM, with studies indicating iron deficiency's role in its physiological and pathological processes [44]. Fig. 4 illustrates the pathogenesis of ferroptosis in T2DM.

#### Lipid peroxidation

Lipid peroxidation, which is caused by the reaction of ROS to attack fragile lipids on the cell membrane, is an important factor in apoptosis [45]. Polyunsaturated fatty acids (PUFA), whose unstable double bonds make them key to ferroptosis and highly susceptible to lipid peroxidation [46]. Studies show PUFAs must be esterified into phospholipids and then oxidized to transmit ferroptosis signals [47]. Notably, lysophosphatidylcholine acyltransferase 3 (LPCAT3) and acyl-CoA oxidase synthetase long-chain family member 4 (ACSL4) play crucial roles in phospholipid remodelling [48]. ACSL4 activates arachidonic acid (AA) and adrenic acid (AdA), participates in fatty acid oxidation and lipid biosynthesis; its products bind CoA to form AA-CoA and AdA-CoA, prerequisites for subsequent lipid remodeling [49]. Next, LPCAT3 catalyzes the transfer of acyl groups (AA or AdA) from AA-CoA/AdA-CoA to the sn-2 position of lysophosphatidylethanolamine, ultimately generating PE-AA (arachidonic acid-containing phosphatidylethanolamine) and PE-AdA [50]. Finally, 15-lipoxygenase (15-LOX) specifically catalyzes the oxidation of PE-AA and PE-AdA, producing abundant lipid peroxides. Their intracellular accumulation disrupts redox balance and triggers ferroptosis [47]. Thus, LPCAT3 and ACSL4 may regulate ferroptosis through PUFA phospholipid metabolism, contributing to T2DM.

#### Long-term exposure to arsenic

Long-term exposure to arsenic is an important risk factor for T2DM [51]. Arsenic reduces insulin synthesis and secretion in pancreatic β-cells, reduces the expression of antioxidant enzymes, and interferes with glucose production in the liver. Therefore, arsenic affects the insulin sensitivity of the surrounding tissues by modifying the expression of genes involved in IR and transferring cells from differentiation to proliferation [52]. Stimulation of AS triggers abnormal release of Cytochrome c (Cytc), which directly damages mitochondria, characterized by a significant decrease in Matrix Metalloproteinases (MMP). Meanwhile, mitochondrial damage induces autophagy, and the autophagic system binds to ferritin via NCOA4-mediated selective autophagy, accelerating the disintegration of iron storage pools, releasing a large amount of free divalent iron ions, inducing the Fenton reaction, and ultimately leading to ferroptosis [53]. Wei et al. [54] used sodium arsenic trioxide (NaAsO_2_) to induce pancreatic dysfunction models in Sprague–Dawley rats and MIN6 cells in vivo and in vitro. They found that ferroptosis was present in both islet β-cell injury models. Mitochondrial damage caused by NaAsO_2_ can lead to excessive production of mitochondrial reactive oxygen species, thereby increasing the concentration of intracellular free iron and promoting mitochondrial reactive oxygen species-dependent autophagy. This chain reaction eventually leads to iron-induced apoptosis and impaired insulin secretion in MIN6 cells.

#### Iron overload

Ferroptosis is directly related to ferritin levels in the body. Early studies [55, 56] have shown the link between iron, IR development, and T2DM. The current idea is that the more iron stored in the body, the higher the risk of T2DM [57, 58]. The intracellular transport and metabolism of Fe^2^⁺ are key to inducing ferroptosis. Free Fe^2^⁺ binds to transferrin, enters the cell via Transferrin Receptor 1 (TFR1), is released in the intracellular acidic environment, and then oxidized to Fe^3^⁺. Cellular iron metabolism imbalance induces ferritinophagy, releasing and overaccumulating Fe^2^⁺. With oxygen, Fe^2^⁺ reacts with H₂O₂ to trigger lipid peroxidation and massive lipid ROS. Exceeding cellular antioxidant capacity damages the membrane, ultimately inducing ferroptosis [59]. It can severely damage pancreatic cells through excessive OS. This impairs the liver's insulin utilization and gluconeogenesis functions, promoting the development and progression of T2DM [60]. Zhang et al. [61] reported that miR-144-3p upregulation suppresses Sirtuin 1 (SIRT1) and USP22, leading to ferroptosis and pancreatic β cell dysfunction, which further accelerates T2DM progression. In a high-glucose environment, diabetes may increase tissue ferritin levels through lipid, iron, and GPX4 metabolism, possibly contributing to T2DM development.

### Pyroptosis

**Fig. 5:**
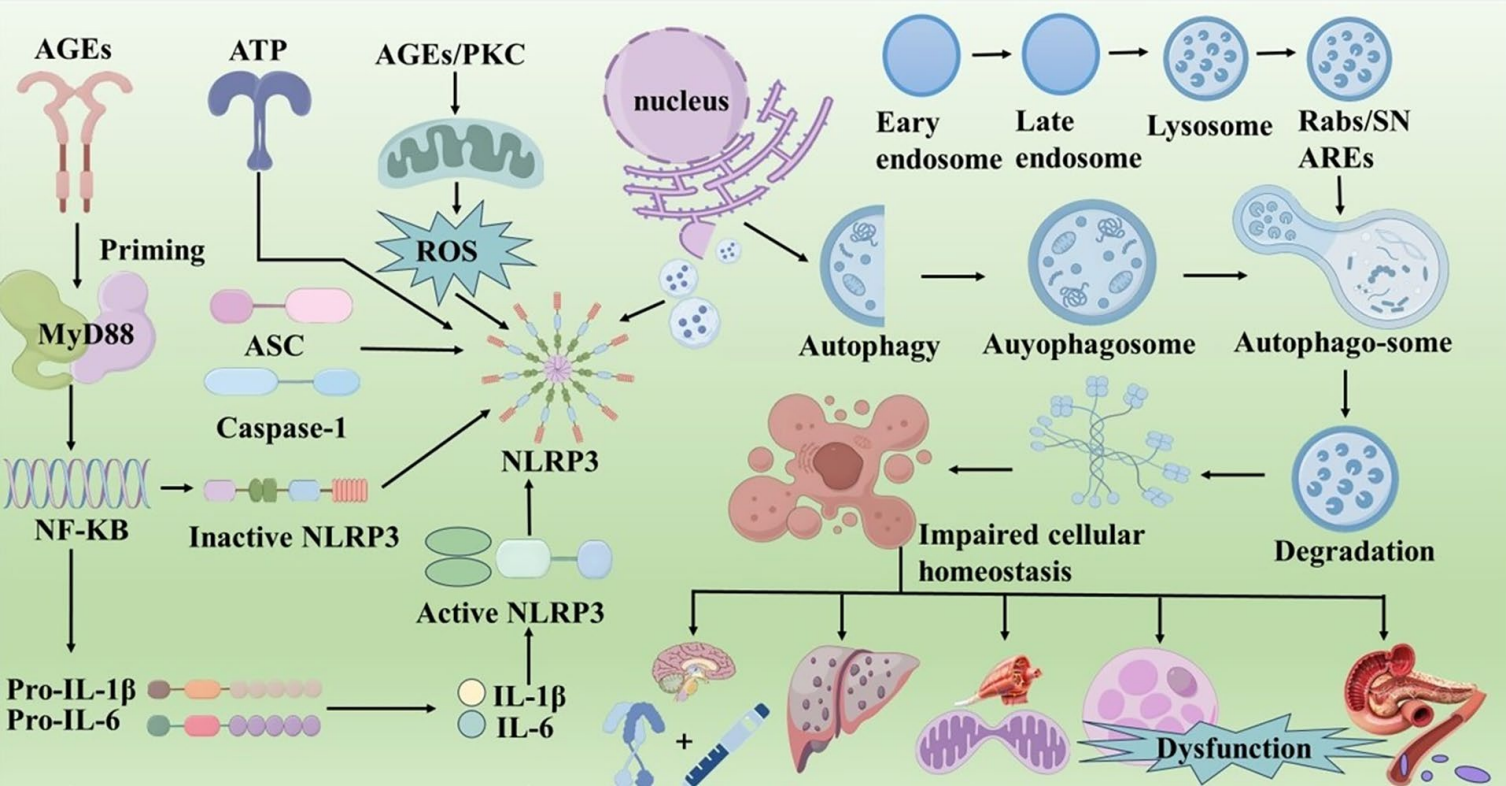
The pathogenic mechanisms of pyroptosis and autophagy in T2DM. NLRP3 is a key member of the NLR family involved in pyroptosis. The NF-κB pathway mediates the transcriptional activation of the NLRP3 inflammasome. Under the influence of PAMPs and DAMPs, adipocyte dysfunction can lead to mitochondrial dysfunction, resulting in the production of ROS. The infiltration of inflammatory cells in adipocytes triggers the secretion of IL-1β and IL-6, which in turn induces systemic inflammation. This cascade further exacerbates abnormal glucose and lipid metabolism and IR, worsening the condition of T2DM.Autophagy occurs in insulin-sensitive tissues such as the hypothalamus, skeletal muscle, liver, and adipocytes, leading to a gradual decline in insulin secretion from the pancreas. Inhibition of autophagy in POMC neurons of the hypothalamus and excessive autophagy in pancreatic β-cells can disrupt insulin signaling and insulin-dependent glucose uptake, thereby increasing the risk of T2DM.Selective autophagy in skeletal muscle (such as mitophagy and lipophagy), hepatic autophagy, and adipocyte autophagy are key factors in the disruption of glucose and lipid metabolism, IR, and the onset of T2DM.

Pyroptosis, an inflammatory programmed cell death mediated by gasdermin proteins (GSDMDs), involves caspase cleavage of gasdermins into N-terminal pore-forming domains and C-terminal repressor domains [62–64]. GSDMD activation relies on inflammasome formation, triggering inflammatory caspases (− 1, − 4, − 5, − 11). Studies have indicated that pyroptosis is closely associated with the pathogenesis of T2DM [65]. The pathogenesis is illustrated in Fig. 5.

#### NOD-like receptor family, pyrin domain containing 3 inflammasome

NOD-like receptor family pyrin domain-containing 3 (NLRP3), a key member of the NLR family, is a central regulator of pyroptosis. The NLRP3 inflammasome consists of three components: the sensor (NLRP3), effector (Caspase-1), and adaptor (ASC) [66]. NLRP3 inflammasome activation requires two signals: the initiation of NLRP3 gene transcription through the NF-κB pathway and the activation of NLRP3 inflammasome sensors [67]. When AGEs initiate the priming process, they bind to pattern recognition receptors and then recruit MyD88. Through a cascade reaction, NF-κB is activated; the activated NF-κB enters the nucleus to promote the expression of NLRP3, pro-IL-1β, and pro-IL-18, thereby reserving key molecules for the activation of the NLRP3 inflammasome [68]. In T2DM, glucose, fatty acids, mitochondrial ROS, ceramides, homocysteine, and adenosine triphosphate can activate the NLRP3 inflammasome, which is mediated by NIMA-related kinase 7 interaction upon stimulation. In 2010, Tschopp et al. [69]first suggested the NLRP3 inflammasome may play a role in T2DM progression. Subsequent studies show NLRP3 inflammasome activation exacerbates IR and damages islet cells, accelerating T2DM progression [70, 71].

#### Dysfunction of adipocytes and chronic inflammation

Studies show hypertrophic and hypoxic adipocytes in obese rats cause mitochondrial dysfunction and ROS production, while reduced antioxidant enzyme activity exacerbates OS [72]. This process activates pyroptosis-related molecules, leading to adipocyte cleavage and death. Concurrently, inflammatory cell infiltration in adipose tissue releases cytokines, including MCP-1/CCL-2, IL-1/IL-6, and TNF-α, triggering systemic inflammation. This cascade worsens glucose and lipid metabolism abnormalities and IR, exacerbating T2DM [73].

#### Chronic hyperglycaemia

Chronic hyperglycemia elevates mitochondrial metabolism via AGEs and PKC, increasing reactive ROS. This triggers pyroptosis, damaging pancreatic β-cells and vascular endothelial cells, and causing inflammation, leading to β-cell dysfunction, IR, and endothelial dysfunction [74]. The activation of thioredoxin-interacting protein in β-cells further promotes cell lysis and death, intensifying the inflammatory cascade [75]. Studies have shown that elevated mitochondrial ROS levels are more pronounced in patients with poorly controlled chronic hyperglycaemia, leading to activation of the pyroptosis pathway and heightened inflammatory response [76]. Additionally, the abnormal deposition of islet amyloid polypeptide (IAPP) in β-cells and pancreatic capillaries during the progression of T2DM not only drives interstitial fibrosis but also exerts direct cytotoxic effects. Researchers have observed that within these IAPP deposits, there is significant activation of pyroptosis-related proteins, marked aggregation of macrophages, and a substantial increase in the release of IL-1β and chemokines. These pathological changes collectively trigger β-cell lysis and initiate sustained inflammatory cascades, which in turn exacerbate the progression of T2DM [77].

### Autophagy

In the process of autophagy, macroautophagy is the most focused type in T2DM. Its core process is as follows: after the formation of double-membraned autophagosomes, they enwrap intracellular components and transport them to lysosomes. Subsequently, the contents are decomposed by lysosomal enzymes, and macromolecular substances are degraded into small molecules, which are then recycled to the cytoplasm for reuse [78, 79]. If abnormalities such as Ras-related in brain GTPases (Rab) inactivation, Soluble N-ethylmaleimide-sensitive factor attachment protein receptors (SNAREs) dysfunction, or insufficient lysosomal enzyme activity occur during this process, it will hinder the formation of autolysosomes or reduce degradation efficiency, thereby triggering cellular homeostasis imbalance, which manifests as cellular senescence, apoptosis, or inflammatory responses and is closely associated with the occurrence and development of T2DM [80]. Furthermore, insulin sensitivity is key to T2DM pathogenesis. Autophagic dysfunction in insulin-sensitive tissues (hypothalamus, skeletal muscle, liver, adipose) impairs their insulin sensitivity, gradually reducing pancreatic insulin secretion and worsening the disease [81]. The mechanism is illustrated in Fig. 5.

#### Hypothalamus

The hypothalamus is crucial for coordinating hormone signalling and balancing energy expenditure. Neurones in the arcuate melanocortin system, particularly those that produce proopiomelanocortin (POMC)-derived peptides, play a significant role in T2DM [82, 83]. Downregulation of Autophagy-Related Gene 7 (ATG7) in POMC neurones impairs autophagy in the hypothalamus, leading to decreased insulin sensitivity, accelerated glucose intolerance, and increased risk of developing T2DM [84]. In addition, the inhibition of autophagy in hypothalamic POMC neurons can lead to the loss of primary cilia, thereby disrupting the localization of insulin receptors within the cilia and ultimately impairing insulin signal transduction and insulin-dependent glucose uptake processes [85].

#### Skeletal muscle

The relationship between IR and impaired autophagy in skeletal muscles has been demonstrated in both human and in vitro studies. For example, in patients with T2DM [86, 87].

The expression of autophagy-related genes is decreased, and these patients require over 100 U of insulin daily, which is associated with abnormal mitochondrial morphology and function in skeletal muscles [88]. Furthermore, palmitate-induced cellular senescence and IR in cultured muscle cells occur due to disruption of autophagic flux [89]. Insufficient or impaired mitophagy may accumulate damaged mitochondria in skeletal muscle [90]. Furthermore, increased lipid deposition in skeletal muscle cells can contribute to age-related declines in mitochondrial function. Lipophagy plays a crucial role in the degradation of lipid droplets, and its impairment may accumulate these droplets, ultimately causing lipotoxicity in skeletal muscle [91]. Therefore, impairments in selective autophagy (such as mitophagy and lipophagy) in skeletal muscle could be key factors in the pathogenesis of age-associated IR and T2DM.

#### Liver

The liver is a central organ that regulates metabolism and maintains energy balance. Hepatic autophagy is now recognised as playing a crucial role in the regulation of glucose and lipid metabolism, IR, and overall energy homeostasis [92]. Under IR conditions, the liver experiences disturbances in glucose and lipid metabolism, leading to hyperglycaemia and hypercholesterolemia [93]. In diet-induced obese and ob/ob mice, intra—brown adipose tissue injection of adenovirus encoding Nrf1 or the proteasome activator PA28α can enhance proteasomal degradation, improving IR [94]. In addition, ablation of the proteasome activator PA28 leads to endoplasmic reticulum stress in the liver, which exacerbates glucose intolerance following liver IR and high-fat diet (HFD) exposure, ultimately disrupting insulin signal transduction [95].

#### Adipose tissue

Adipose tissue is crucial for regulating the body's energy and glucose balance and is closely linked to insulin sensitivity control [96]. Atg4b^−/−^ mice on a high-calorie diet, with partial autophagy deficiency, became obese and had enlarged adipocytes [97]. This shows that autophagy's adaptive response to metabolic stress can regulate metabolic diseases such as T2DM. Autophagy in subcutaneous and visceral adipose tissue is upregulated in patients with obesity or T2DM [98–100]. With the increase of age, the expression of Rubcn in adipose tissue decreased, and the activity of autophagy was enhanced. Mouse Rubcn adipocyte-specific knockout experiments showed that fat atrophy, poor glucose tolerance, dyslipidemia, and liver fat accumulation were associated with decreased lipid storage capacity and decreased endocrine function of adipokines [101]. Thus, Rubicon protein is essential for maintaining adipocyte function and systemic metabolic balance by inhibiting excessive autophagy.

#### Pancreas

Pancreatic β cells are vital for insulin production, storage, and secretion. Studies indicate that β-cell-specific knockout of ATG7 in mice impairs autophagy, resulting in elevated β-cell death and reduced β-cell proliferation [102]. Studies have shown that pancreatic β cells in T2DM patients exhibit more abundant autophagic vacuoles and autophagosomes than β cells in non-diabetic patients, and the expression of lysosomal genes is lower, indicating that T2DM leads to changes in the level of autophagy structure removal [103]. The simultaneous activation of autophagy triggered by intracellular stress, such as endoplasmic reticulum stress and blockage of autophagic flux, can cause the accumulation of defective lysosomes. This accumulation ultimately leads to the death of β-cells and the onset of T2DM [104].

### Gut microbiota

**Fig. 6:**
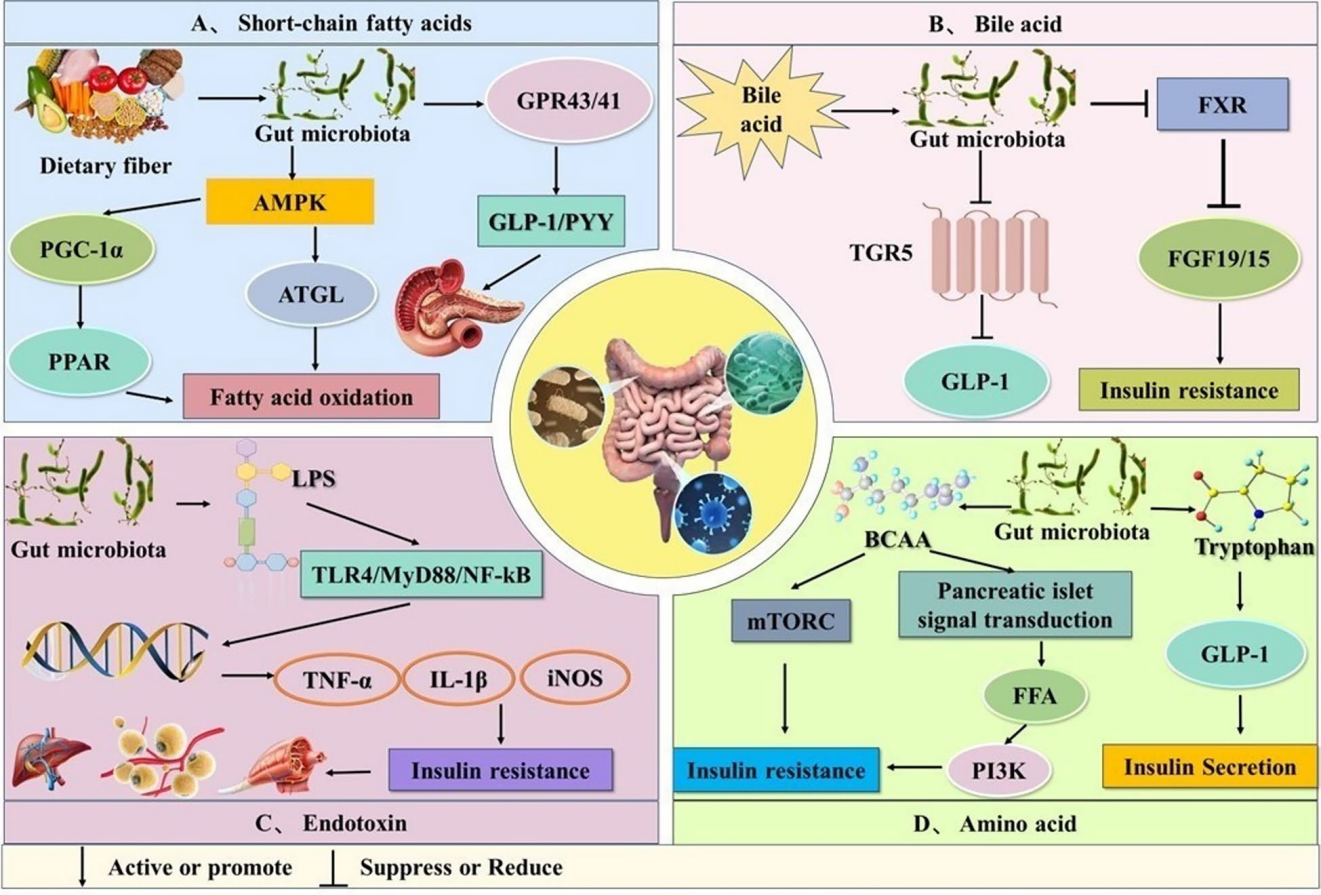
Pathogenic mechanisms of gut microbiota in T2DM. (**A**) illustrates the theory of short-chain fatty acids.After dietary fiber is metabolized in the intestine, acetate is produced, which binds to GPR41/43, enhancing AMPK phosphorylation and thus affecting fatty acid oxidation and glucose metabolism. GPR41 inhibits insulin secretion from pancreatic β-cells and PYY secretion from intestinal L-cells, leading to increased food intake and the development of IR. GPR43 obstructs adipose tissue signaling, reducing energy expenditure, increasing body fat, and promoting apoptosis in pancreatic β-cells, which decreases GLP-1 secretion and lowers insulin secretion and sensitivity. The AMPK pathway can regulate molecules such as PGC-1α, PPAR, and ATGL, participating in fatty acid oxidation and being closely associated with the development of T2DM. (**B**) illustrates the bile acid theory. Bile acids, as steroid molecules, are synthesized in the liver and converted from primary bile acids to secondary bile acids by hydrolytic enzymes and 7α-dehydroxylase. This process promotes the activation of FXR, leading to a decrease in the secretion of FGF19 and FGF15D, which in turn causes weight gain, reduced energy expenditure, and decreased insulin sensitivity. After dysbiosis, the secretion of both primary and secondary bile acids is suppressed, and the TGR5 pathway is activated, resulting in reduced secretion of GLP-1, lower insulin levels, and increased glucagon levels. This leads to increased gastric emptying and appetite, further reducing insulin sensitivity and triggering the onset of T2DM. (**C**) illustrates the endotoxin theory, with LPS being a key type of endotoxin. When the gut microbiota is disrupted, resulting in an increase in Gram-negative bacteria, LPS levels also rise. LPS activates the downstream pathways of TLR4, including MyD88 and TRAF, leading to the excessive expression of pro-inflammatory factors such as TNF-α, IL-1β, iNOS, and IL-6, which affect the metabolism of skeletal muscle, adipose tissue, and the liver. Ultimately, the excess LPS enters the bloodstream, causing endotoxemia, stimulating inflammation, and inhibiting insulin signaling pathways and insulin sensitivity. This results in increased IR, triggering the onset of T2DM. (**D**) illustrates the amino acid theory. Bioactive small molecules produced by gut microbiota metabolism, such as branched-chain amino acids and aromatic amino acids, are closely related to IR. Branched-chain amino acids can induce the (mTORC signaling pathway and disrupt normal insulin signaling, leading to increased FFA, activation of PI3K, and thereby inducing IR. Another aromatic amino acid, tryptophan, can stimulate GLP-1 release from intestinal L cells while promoting insulin secretion from pancreatic β-cells, contributing to the development of T2DM.

Healthy adults host about 100 trillion gut microorganisms, ten times the number of human cells, forming a "second genome" with over 35,000 bacterial species [105, 106]. Gut microbiota dysbiosis, along with obesity, genetics, and insulin dysfunction, is a key contributor to T2DM [107]. It is noteworthy that some genes in patients with T2DM have been altered. The dysregulated expression (either upregulation or downregulation) of T2DM-associated genes may induce DNA methylation [108] and perturb pathways such as glycolysis [109], and gluconeogenesis [110]. These perturbations disrupt the structural integrity of glucose homeostasis and alter IR, ultimately elevating fasting blood glucose levels and contributing to the development of T2DM. Additionally, the gut microbiota and their metabolites can impair pancreatic islet function via mechanisms involving short-chain fatty acids, bile acid metabolism, and endotoxin-mediated responses. This impairment not only reduces insulin sensitivity but also disrupts glucose and lipid metabolism, thereby exacerbating metabolic dysregulation. The pathogenesis is illustrated in Fig. 6.

#### Short-chain fatty acid theory

Short-chain fatty acids (SCFA) are mainly produced by glycolysis and the fermentation of carbohydrates that escape digestion and absorption in the small intestine. These are butyrate, propionate, and acetate, respectively [111, 112]. In T2DM, reduced butyrate-producing microbiota (e.g., *Roseburia*, *Faecalibacterium prausnitzii*) lowers intestinal butyrate, impairing GPR43/41-mediated GLP-1 and Peptide YY (PYY) secretion. This causes defective insulin secretion and exacerbated glycemic fluctuations, driving T2DM progression [113, 114]. Propionate can also phosphorylate PGC-1α via the AMPK signaling pathway, enhancing its binding capacity to PPARα and forming a "receptor-coactivator" functional complex [115]. Acetate, by binding to GPR43, can upregulate the phosphorylation level and activity of AMPK, thereby promoting the release of adipose triglyceride lipase (ATGL), and ultimately affecting the process of fatty acid oxidation [116]. Advanced sequencing revealed that 345 Chinese T2DM patients had reduced levels of butyric acid-producing *Roseburia intestinalis* and *Faecalibacterium prausnitzii*, but elevated levels of *Lactobacillus gasseri*, *Proteus*, and some *Clostridium* species [117, 118]. Thus, disruption of intestinal homeostasis can alter the levels of SCFAs, which in turn impacts host energy metabolism and contributes to the development of IR and T2DM.

#### Bile acid theory

Bile acids (BA), cholesterol-derived steroid molecules synthesized in the liver, are essential for regulating glucose metabolism and energy balance in T2DM patients [119]. Bile acid hydrolases and 7α-dehydrogenases in the gut microbiota metabolize liver-produced primary bile acids into secondary bile acids, FXR (Farnesoid X Receptor) and TGR5 [120, 121]. Dysbiosis of the gut microbiota reduces the activity of bile salt hydrolase and 7α-dehydroxylase, leading to a decrease in free primary bile acids and an abnormal proportion of secondary bile acids. This restricts the activation of FXR, thereby reducing the secretion of FGF19/15. Under such circumstances, hepatic gluconeogenesis is enhanced and lipid accumulation is aggravated, which induces IR [122]. Furthermore, gut dysbiosis reduces the activity of bile salt hydrolase and 7α-dehydrogenase, leading to decreased levels of secondary bile acids (e.g., lithocholic acid) and insufficient activation of TGR5. This directly inhibits the secretion of GLP-1 by L cells, and such dysregulation may exacerbate the progression of metabolic diseases such as T2DM [123]. However, long—term FXR activation shrinks the bile acid pool, reducing energy use, increasing IR, and contributing to obesity and T2DM [124]. Imbalances in gut microbiota and bile acid metabolism disrupt glucose metabolism pathways, causing disordered glucose and lipid metabolism and worsening T2DM progression.

#### Endotoxin theory

Lipopolysaccharide (endotoxin), a key component of gram-negative bacterial cell walls, triggers immune activation and strong inflammatory responses [125]. In *vitro* and in *vivo* evidence indicates TLRs mediate lipopolysaccharide (LPS) induced inflammatory responses [126]. Dysbiosis of the gut microbiota increases the production of lipopolysaccharides, activates the TLR4/MyD88/NF-kB pathway, and elevates pro-inflammatory cytokines (IL-1β, IL-6, TNF-α), which induces IR and thereby affects tissues such as muscle, adipose, and liver [127]. Furthermore, microbiota imbalance reduces diversity, increases gut permeability, and promotes LPS entry into the bloodstream, causing endotoxemia. Studies show reduced intestinal butyrate, a metabolite of *Akkermansia muciniphila*. Endotoxins activate CCR^2+^ monocytes, which infiltrate the omentum, convert B1a lymphocytes to 4-1BBL-expressing B cells, and induce hyperglycemia and IR via 4-1BB Ligand expression [128]. LPS produced by the gut microbiota participates in the host’s glucose and lipid metabolism, which produces an inflammatory response that triggers T2DM.

#### Others

Branched-chain amino acids (BCAAs), aromatic amino acids, triglycerides, and trimethylamine oxide are closely associated with IR [129–131]. The mechanism of BCAA-induced IR is closely linked to the mammalian target of rapamycin complex (mTORC) signalling pathway [132]. BCAAs can block the normal conduction of insulin signals, increase the oxidation of free fatty acid, activate PI3K, and induce IR [133]. Tryptophan, one of the essential aromatic amino acids in the human body, has metabolites such as indoxyl sulfate and p-cresol sulfate that can stimulate the release of GLP-1 and insulin secretion. These actions contribute to lowering blood glucose, regulating hepatic oxidative stress, reducing intestinal inflammation, and improving islet cell morphology [134, 135]. Moreover, trimethylamine oxide and imidazole propionate affect insulin signal transduction by acting on different pathways [136]. But due to scarce related research, more studies are required to clarify the potential mechanism between gut microbiota metabolites and T2DM.

## The ameliorating effects of TCM formulas on T2DM

TCM is widely used in clinical disease prevention and treatment, with ample Chinese clinical evidence confirming its significant role in improving and adjuvant treating T2DM [137]. According to TCM theory, the core pathogenesis of T2DM encompasses yin deficiency with dryness-heat, qi-yin deficiency, blood stasis, and phlegm-dampness accumulation. Key therapeutic principles are clearing heat-moistening dryness, nourishing yin-promoting fluid, replenishing qi-nourishing yin, activating blood-resolving stasis, and resolving phlegm-eliminating dampness. In syndrome classification studies, these "four syndromes" are most common: qi-yin deficiency, yin deficiency with dryness-heat, blood stasis, and phlegm-dampness [138]. These findings highlight both the importance of the "four syndromes" as T2DM's fundamental pathological mechanisms and the key value of clearing heat-moistening dryness, nourishing yin-promoting fluid, replenishing qi-nourishing yin, activating blood-resolving stasis, and resolving phlegm-eliminating dampness as its main treatments.

In recent years, extensive academic research has aimed to identify evidence-based Chinese herbal formulas for treating T2DM. These formulas have gained recognition among the general public and small research institutions due to their proven efficacy. This chapter summarizes in vitro, in vivo, and clinical studies of such formulations in the past five years (Table 1).

**Table 1:** Traditional Chinese medicine compound preparations for the treatment of T2DM in 2020 to 2025

No	TCM formula	Prescription composition	Types	Dosages, route,	Models	Effects	References
1	Zengye Decoction	*Scrophularia ningpoensis* Hemsl. (Xuan Shen)*, Ophiopogon japonicus* (Thunb.) Ker Gawl (Mai Dong)*, and Rehmannia glutinosa* (Gaertn.) DC. (Sheng Di Huang)	In vivo In vitro	4, 8, 16 g/kg daily for 3 weeks; 25, 50 and 100 μg /mL for 24 h	HFD/STZ-induced T2DM model; IR-HepG2 induced by palmitic acid, IR-C2C12 myotubes, and IR-3T3-L1 adipocytes	HDL-C↑, TC↓, TG↓, LDL-C↓, Phosphorylation level of Akt↑, GLUT 4↑, FBG↓	[2]
2	Gegen Qinlian Decoction	*Pueraria lobata* (Willd.) Ohwi (Ge Gen)*, Scutellaria baicalensis* Georgi (Huang Qin)*, Coptis chinensis* Franch. (Huang Lian)*, and Glycyrrhiza uralensis* Fisch. (Zhi Gan Cao)	In vivo	25 g/kg of GQD concentrate at a volume of 5 mL/kg and a concentration of 5 g/mL daily for 8 weeks	HFD/STZ-induced T2DM model	TC↓, TG↓, LDL-C↓, HDL-C↑, *Lactobacillus*↑, *colibacillus*↓, *Coprococcus*↑, *Bifidobacterium*↑, *Blautia*↑, *Akkermansia*↑	[14]
3	Huanglian Jiedu Decoction	*Coptis chinensis* Franch. (Huang Lian)*, Scutellaria baicalensis* Georgi (Huang Qin)*, Phellodendron amurense* Rupr. (Huang Bai)*, and Gardenia jasminoides* J.Ellis (Zhi Zi)	In vivo	3, 1.5 and 0.75 g/kg daily for 9 weeks	HG/HPD/STZ -induced T2DM cognitive dysfunction animal model	IL-1β↓, ATG 7↑, LC 3 proteins↑, P62↓, NLRP 3↓, caspase-1↓	[146]
4	Yuye Decoction	*Astragalus membranaceus* Moench (Huang Qi)*, Dioscorea opposita Thunb. (Shan Yao), Anemarrhena asphodeloides* Bunge (Zhi Mu)*, Pueraria lobata* (Willd.) Ohwi (Ge Gen)*, Trichosanthes kirilowii* Maxim. (Tian Hua Fen)*,* *Schisandra chinensis* (Turcz.) Baill. (Wu Wei Zi)*, Endothelium corneum gigeriae galli* (ji Nei Jin)	In vivo In vitro	0.725 and 2.9 g/kg daily for 8 weeks; 12.5, 25, 37.5, 50, 75, 100, 150, 200 μg/mL were incubated in dark for 2 h	HG/HPD/STZ -induced T2DM model; HG and PA induced Min 6 cell apoptosis model	Bax/Bcl-2↓, cleaved-Caspase 3↓, Sirt 1↓, p-FoxO 1↓, FoxO 1↑, PI3K/AKT↑	[154]
5	Da-Chai-Hu Decoction	*Bupleurum chinense* Franch. (Chai Hu)*, Scutellaria baicalensis* Georgi (Huang Qin)*, Pinellia ternata* Ten. ex Breitenb. (Ban Xia)*, Paeonia lactiflora* Pall. (Shao Yao)*, Citrus aurantium* L. (Zhi Shi)*, Rheum palmatum* L. (Dai Huang)*, Zingiber officinale* Roscoe (Sheng Jiang)*, Ziziphus jujuba* Mill. (Da Zao)	In vivo	10.1 g/kg every day	HG/HPD/STZ -induced T2DM model	FBG↓, HbAlc↓, FFA↓, TC↓, MDA↓, ROS↓, SOD↑, PDX-1 mRNA↑, MafA mRNA↑	[161]
6	Jinlida granules	*Panax ginseng* C.A.Mey. (Ren Shen)*, Polygonatum sibiricum* F.Delaroche (Huang Jing), *Atractylodes lancea* (Thunb.) DC.(Cang Zhu), *Sophora flavescens* Aiton (Ku Shen), *Ophiopogon japonicus* (Thunb.) Ker Gawl (Mai Dong)*, Rehmannia glutinosa* (Gaertn.) DC. (Sheng Di Huang), *Polygonum multiflorum* Thunb.(He Shou Wu), *Lycium barbarum* L.(Di Gu Pi), *Litchi chinensis* Sonn.(Li Zhi He), *Pueraria lobata* (Willd.) Ohwi (Ge Gen), *Salvia miltiorrhiza* Bunge (Dan Shen), *Epimedium brevicornu* Maxim.(Yin Yang Huo) and *Anemarrhena asphodeloides* Bunge(Zhi Mu)	In vivo	3.8 g/kg daily for 15 weeks	HFD-fed C57BL/6 J mice induced metabolic disorder model	TAG↓, LDL-C↓, Lipids of NEFA ↓, PRDM16↑, DIO2↑, ELOVL3↑, PGC-1α↑, UCP1↑	[165]
7	Shenqi Jiangtang Granules	*Panax ginseng* C.A.Mey. (Ren Shen)*, Astragalus membranaceus M*oench (Huang Qi), *Rehmannia glutinosa* (Gaertn.) DC. (Sheng Di Huang), *Ophiopogon japonicus* (Thunb.) Ker Gawl (Mai Dong)*, Trichosanthes kirilowii* Maxim. (Tian Hua Fen)*,* *Lycium barbarum* L.(Gou Qi), *Schisandra chinensis* (Turcz.) Baill.(Wu Wei Zi), *Rubus idaeus* L.(Fu Peng Zi), *Dioscorea opposita* Thunb.(Shan Yao), *Wolfiporia cocos* (Schwein.) Ryvarden & Gilb.(Fu Ling) and *Alisma orientale* (Sam.) Juzep.(Ze Xie)	In vivo	400 and 800 mg / kg daily for 6 weeks	HFD induced IR model in rats	P-NFκB↓, TNF-α↓, IL-6↓, IL-1β↓, Akt phosphorylation↑, GLUT2↑	[172]
8	Liuwei Dihuang Pills	*Rehmannia glutinosa* (Gaertn.) DC. (Shu Di Huang)*, Cornus officinalis* Siebold & Zucc. (Shan Zhu Yu)*, Dioscorea opposita* Thunb. (Shan Yao)*, Alisma orientale* (Sam.) Juz. (Ze Xie)*, Wolfiporia extensa* (Peck) Ginns (Fu Ling) *and Paeonia suffruticosa* Andrews (Mu Dan Pi)	In vivo	0.67 g/ml, and 1 mL/ per 100 g body-weight daily for 12 weeks	STZ-induced diabetic rat model	TGFb/SMAD↓, MAPK↓, NF-kB↓, CYGB↑, SOD↓, MDA↑,	[288]
9	Yuquan Pill	*Pueraria lobata* (Willd.) Ohwi (Ge Gen)*, Rehmannia glutinosa* (Gaertn.) DC. (Di Huang)*, Ophiopogon japonicus* (Thunb.) Ker Gawl (Mai Dong)*, Trichosanthes kirilowii* Maxim. (Tian Hua Fen)*, Schisandra chinensis* (Turcz.) Baill. (Wu Wei Zi)*, and Glycyrrhiza uralensis* Fisch. (Gan Cao)	In vivo	2.16 g/kg daily for 5 weeks	HFD/STZ-induced T2DM model	*Firmicutes*↓, Bacteroidetes↑, *Ruminococcus*↓, *lactobacillus*↑, *citrobacter amalonaticuscitrobacter diversus*↓	[182]
10	JinQi Jiangtang tablets	*Coptis chinensis* Franch. (Huang Lian)*, Astragalus membranaceus* Moench (Huang Qi) *and Lonicera japonica* Thunb. (Jin Yin Hua)	In vivo	0.655 and 1.3 g/kg daily for 8 weeks	HFD/STZ-induced T2DM model	FBG↓, TG↓, PI3K-AKT↓, IRS-1↑, GLUT 1↑, GLUT 2↑	[187]

### Zengye decoction (ZYD)

Zengye Decoction (ZYD) is a renowned TCM formula, originating from *Treatise on Febrile Diseases* (Volume II) written by Wu Jutong in the Qing Dynasty. It is commonly used to alleviate and manage symptoms of "Xiao ke" syndrome, which is analogous to T2DM. ZYD consists of three Chinese herbs: *Scrophularia ningpoensis* Hemsl*.* (Xuan Shen), *Ophiopogon japonicus* (Thunb.) Ker Gawl (Mai Dong), and *Rehmannia glutinosa* (Gaertn.) *DC.* (Sheng Di Huang). Among them, *Scrophularia ningpoensis* Hemsl*.* (Xuan Shen) is salty in taste and cold in nature, with the effect of nourishing yin; *Ophiopogon japonicus* (Thunb.) Ker Gawl (Mai Dong) is sweet in taste and cold in nature, capable of promoting fluid production; *Rehmannia glutinosa* (Gaertn.) *DC.* (Sheng Di Huang) has a bitter, astringent and sweet taste, and possesses the effect of clearing heat. The combination of these three herbs not only relieves intestinal dryness and constipation caused by yin-fluid deficiency, but also improves the symptoms of "Xiao ke" by nourishing yin and reducing fire [2]. Modern pharmacological studies have shown that ZYD can be used for the treatment of T2DM [2]. Studies indicate that ZYD's active ingredients lower serum levels of pro-inflammatory cytokines (TNF-α, IL-1β, IL-6, IL-17), modulate Bcl-2 expression, and provide anti-inflammatory and antioxidant effects [139]. Compared to the untreated T2DM group, ZYD at various doses increased high-density lipoprotein cholesterol (HDL-C) levels, reduced serum total cholesterol (TC), low-density lipoprotein cholesterol (LDL-C) and triglyceride (TG) levels, improved islet cell sensitivity, and exhibited significant hypoglycaemic effects in T2DM treatment [2]. In addition, ZYD reduced levels of potentially pathogenic bacteria like *Bacteroides, Clostridium vadin BB60*, and uncultured *Clostridium*, while enhancing SCFA production by beneficial bacteria such as *Erysipelothrix, Bifidobacterium,* and *Lactobacillus* [140]. These findings suggest that ZYD may offer therapeutic potential for T2DM by reducing harmful bacteria and modulating gut microbiota.

### Gegen Qinlian Decoction (GQD)

Gegen Qinlian Decoction (GQD), from Zhang Zhongjing's *Treatise on Febrile Diseases* (Eastern Han Dynasty), aligns with TCM pathogenesis of diabetes like "yin deficiency with dryness-heat", "qi-yin deficiency" and "internal dampness-heat", with its core application targeting "intestinal dampness-heat syndrome". It is composed of four herbal components, namely *Pueraria lobata* (Willd.) Ohwi (Ge Gen), *Scutellaria baicalensis* Georgi (Huang Qin), *Coptis chinensis* Franch. (Huang Lian), and *Glycyrrhiza uralensis* Fisch. (Zhi Gan Cao) [141]. *Pueraria lobata* (Willd.) Ohwi (Ge Gen) alleviates "xiao ke" (wasting-thirst) by promoting fluid and quenching thirst; *Scutellaria baicalensis* Georgi (Huang Qin) and *Coptis chinensis* Franch. (Huang Lian) clear intestinal dampness-heat to target the root; *Glycyrrhiza uralensis* Fisch. (Zhi Gan Cao) harmonizes spleen and stomach. Together, they clear heat, dry dampness, promote fluid and relieve thirst—dispelling dampness-heat, restoring fluids, and reducing "xiao ke", it has been widely adopted in clinical settings [142]. Ren et al. [143] demonstrated that GQD could help mitigate elevated blood glucose levels by improving pancreatic islet function while reducing IR and modulating insulin secretion, with no observed adverse effects. It decreases fasting blood glucose, fasting insulin, glycosylated haemoglobin, ceramide, bile salt hydrolase, and liver mitochondrial acetyl coenzyme A in T2DM rats. Molecularly, GQD suppresses ileal FXR, Serine palmitoyltransferase long-chain subunit 2, and sphingomyelin phosphodiesterase 3 expression and reduces liver OS markers [144]. GQD enhances rat growth, reduces lipids and enriches beneficial gut bacteria like *faecal cocci*, *bifidobacteria*, *Blautia*, and *Akkermansia* [14]. Moreover, a report involving 17 studies with a total of 1,476 patients showed that GQD combined with conventional treatment can significantly improve glycolipid metabolism and IR in patients with T2DM, although its efficacy when used alone remains unclear, it is expected to become an effective anti-IR drug [145].

### Huanglian jiedu decoction (HLJDD)

Huanglian Jiedu Decoction (HLJDD), a renowned TCM heat-clearing and toxin-resolving formula, first appeared in Ge Hong's *Emergency Prescriptions Kept in the Elbow* (Volume 2) [146]. It includes four herbs: *Coptis chinensis* Franch. (Huang Lian), *Scutellaria baicalensis* Georgi (Huang Qin), *Phellodendron amurense* Rupr. (Huang Bai), and *Gardenia jasminoides* J.Ellis (Zhi Zi) [147]. *Coptis chinensis* Franch. (Huang Lian) clears middle-jiao heat-toxin, *Scutellaria baicalensis* Georgi (Huang Qin) upper-jiao lung heat, *Phellodendron amurense* Rupr. (Huang Bai) lower-jiao dampness-heat, and *Gardenia jasminoides* J.Ellis (Zhi Zi) purges triple-jiao fire. Together, they clear heat without damaging yin, resolve toxin and eliminate dampness, improving "heat disturbing fluids" and "dampness obstructing qi" to alleviate xiao ke symptoms like thirst, irritability and reddish urine. Applicable to early-to-middle diabetes with marked heat syndrome. HLJDD has been clinically used in China for the prevention and management of T2DM and is officially listed in the Chinese Pharmacopoeia [148].He et al. [149] BBR and Bai target dipeptidyl peptidase-4, cysteine-aspartic acid protease 3, nitric oxide synthase 2, nitric oxide synthase 3, and MAPK/HIF-1 pathways. Endogenous metabolite changes (e.g., l-valine, l-sorbitol) regulate glucose/lipid metabolism, improve IR, inhibit apoptosis, and exert antioxidant and anti-inflammatory effects, ultimately contributing to the alleviation of T2DM. In an HFD/STZ-induced T2DM model, HLJDD (0.42 g/kg/d or 1.25 g/kg/d) for 8 weeks reduced Malondialdehyde (MDA) and IL-6 levels while increasing Superoxide Dismutase (SOD), demonstrating enhanced antioxidant and anti-inflammatory effects [150]. HLJDD increased beneficial bacteria (*Parabacteroides*, *Blautia*, *Akkermansia*) and reduced pathogens (*Aerococcus*, *Staphylococcus*, *Corynebacterium*), enhancing gluconeogenesis, and nucleotide metabolism to restore gut microbiota function in T2DM [151]. This meta-analysis of 40 studies (3,934 participants) showed HLJDD, alone or combined, significantly improves blood glucose, IR and lipids in T2DM; short-term (< 3 months) monotherapy may better reduce HbA1c and 2-h postprandial glucose, with good safety, supporting it as an effective, safe alternative therapy for T2DM [152].

### Yuye decoction (YYD)

Yuye Decoction (YYD) takes "replenishing qi to promote fluid production and nourishing yin to moisten dryness" as its core therapeutic principle. Targeting the qi-yin deficiency syndrome in "xiao ke", it regulates qi and yin, restores the transportation and distribution of body fluids, thereby becoming an important formula in TCM for treating "xiao ke". Its composition includes *Astragalus membranaceus* Moench (Huang Qi), *Dioscorea opposita* Thunb. (Shan Yao), *Anemarrhena asphodeloides* Bunge (Zhi Mu), *Pueraria lobata* (Willd.) Ohwi (Ge Gen), *Trichosanthes kirilowii* Maxim. (Tian Hua Fen), *Schisandra chinensis* (Turcz.) Baill. (Wu Wei Zi), *Endothelium corneum gigeriae galli* (ji Nei Jin)[153]. *Astragalus membranaceus* Moench (Huang Qi), the sovereign herb, replenishes qi to elevate yang and promote fluid distribution, fundamentally resolving "fluid stagnation". *Dioscorea opposita* Thunb. (Shan Yao), the minister herb, nourishes spleen yin, consolidates kidney and astringes fluids, targeting "yin deficiency" and "profuse urination". Together, they replenish qi, nourish yin, distribute fluids and moisten dryness. YYD active components, including monolinolein, pelargonidin-3-O-glucoside, and acacetin, strongly bind to targets PIK3R1, AKT1, SIRT1, and FoxO1, which are key to glucose metabolism and insulin signalling [154]. YYD reduced the levels of serum lactate dehydrogenase, creatine kinase, and creatine kinase—muscle/brain; reduced the expression of myocardial Bax, IL-6, and TNF-α; and increased P-PI3K and P-AKT levels. YYD may also prevent islet dysfunction and reverse islet β-cell apoptosis through PI3K the AKT1 and SIRT1/FoxO1 signalling pathways [153]. Additionally, YYD has shown certain anti-inflammatory and antioxidant activities in vitro experiments or animal models, and through the regulation of tryptophan metabolism and glycerophospholipid metabolism, it can exert an improving effect on the pathological process of T2DM [155].

### Dachaihu decoction (DCHD)

Dachaihu Decoction (DCHD) comes from *Treatise on Febrile and Miscellaneous Diseases* by "Medical Sage" Zhang Zhongjing [156]. It consists of *Bupleurum chinense* Franch. (Chai Hu), *Scutellaria baicalensis* Georgi (Huang Qin), *Pinellia ternata* Ten. ex Breitenb. (Ban Xia), *Paeonia lactiflora* Pall. (Shao Yao), *Citrus aurantium* L. (Zhi Shi), *Rheum palmatum* L. (Dai Huang), *Zingiber officinale* Roscoe (Sheng Jiang), *Ziziphus jujuba* Mill. (Da Zao) [157]. Sovereign herbs *Bupleurum chinense* Franch. (Chai Hu) and *Scutellaria baicalensis* Georgi (Huang Qin) regulate the Shaoyang pivot; minister herbs *Citrus aurantium* L. (Zhi Shi) and *Ziziphus jujuba* Mill. (Da Zao) expel heat via excretion to prevent fluid consumption. The formula reconciles Shaoyang to smooth qi and purges Yangming to clear heat accumulation, thus relieving xiao ke symptoms like polyphagia, constipation and irritability from heat-consuming fluids and qi stagnation. Network pharmacology experiments have shown that the efficacy markers of naringin, hesperidin, neohesperidin, baicalin, wogonoside, baicalein, and saikosaponin B2 are involved in DCHD [157]. These markers can also inhibit OS and inflammation [158–160]. In vitro studies indicate that under experimental conditions, DCHD may exert a regulatory effect on T2DM-related pathological indicators—specifically by reducing OS levels, promoting the maintenance of β-cell functional stability, and producing a certain relieving effect on fatigue and dehydration symptoms associated with T2DM [161]. Results from 17 randomized controlled trials with 1,552 patients showed that DCHD combined with conventional treatment outperformed conventional treatment alone in improving HbA1c, FBG, 2-h blood glucose, blood lipids, HOMA-IR, HOMA-β and Body Mass Index; when used alone, it had certain improving effects on some blood glucose indicators and was relatively safe [162].

### Jinlida granules (JLDG)

Jinlida Granules (JLDG) is a TCM preparation designed based on the TCM theory of spleen deficiency for the treatment of T2DM with qi-yin deficiency pattern [163]. This Chinese patent medicine consists of 17 herbal ingredients, including *Panax ginseng* C.A.Mey. (Ren Shen), *Astragalus membranaceus* Moench (Huang Qi), *Polygonatum sibiricum* F.Delaroche (Huang Jing), etc. Sovereign herbs *Panax ginseng* C.A.Mey. (Ren Shen) and *Astragalus membranaceus* Moench (Huang Qi) replenish qi to strengthen the spleen, resolving "qi deficiency-induced fluid stagnation". Minister herbs *Polygonatum sibiricum* F.Delaroche (Huang Jing) and *Ophiopogon japonicus* (Thunb.) Ker Gawl (Mai Dong) nourish yin to moisten dryness, relieving "yin deficiency and dryness-heat". The formula replenishes spleen qi, nourishes spleen yin, aids digestion, unblocks collaterals, enables fluid distribution and dryness relief, improving metabolic disorders from "spleen deficiency with qi-yin deficiency" in diabetes. JLDG significantly lowered blood glucose, TG, and LDL levels, increased Uncoupling Protein 1 expression, and reduced obesity in db/db mice by inhibiting the expression of miR-27a in X9 cells [164]. Activating brown adipose tissue thermogenesis is crucial for energy expenditure and treating obesity and T2DM. In a study by Zhang et al. [165] oral administration of JLDG (3.8 g/kg) for 15 weeks ameliorated metabolic dysfunction in HFD-induced obese mice. Data from existing clinical studies suggest that, among the included study participants, JLDG exerts a certain downward-regulating effect on blood glucose levels and can produce varying degrees of alleviating effects on some symptoms associated with patients with T2DM, such as thirst, fatigue, polyuria, dry mouth, spontaneous sweating, night sweats, and constipation [166]. Interestingly, data from 1,810 patients indicated JLDG reduced body mass index, improved β-cell function and IR, with no significant adverse reactions [167]. Based on these results, JLDG exhibits promising antidiabetic efficacy.

### Shenqi Jiangtang granules (SQJTG)

Shenqi Jiangtang granules (SQJTG) is a well-known TCM formulation clinically used to help mitigate elevated blood glucose levels [168]. SQJTG is composed of 11 medicinal herbs based on the “Jun-Chen-Zuo-Shi” principle, ensuring a balanced therapeutic effect. Its sovereign herbs *Panax ginseng* C.A.Mey. (Ren Shen) and *Astragalus membranaceus* Moench (Huang Qi) together replenish qi and strengthen the spleen. Minister herbs *Rehmannia glutinosa* (Gaertn.) *DC.* (Di Huang) and *Cornus officinalis* Siebold & Zucc. (Shan Zhu Yu) synergistically nourish kidney yin and consolidate kidney qi to astringe fluids. It treats diabetes with qi-yin deficiency and spleen-kidney insufficiency via multi-target regulation of qi-blood and nourishment of zang-fu organs, which is approved by the State Food and Drug Administration of China [169]. Clinically, SQJTG has shown positive effects in treating T2DM, especially in relieving symptoms like thirst, polydipsia, polyuria, and fatigue [170, 171]. Network pharmacology shows its active compounds include ginsenosides, verbascoside, etc.[168]. Research indicates SQJTG reduces IL-1, TNF-α, etc. levels, inhibits p-NF/κB overexpression for anti-inflammatory effects. It also upregulates the expression of Akt protein and GLUT2 protein in hepatocytes, thereby restoring the hepatic insulin signaling pathway [172]. In summary, based on existing research findings, SQJTG can exert a certain improving effect on the pathological process of T2DM through the multi-target regulation of neuroendocrine dysfunction, IR, OS, and inflammatory pathways.

### Liuwei dihuang pills (LWDHP)

Liuwei Dihuang Pill (LWDHP) is a TCM compound preparation first recorded in *Key to Children's Medication* of the Song Dynasty, used for the treatment and prevention of chronic metabolic diseases [173, 174]. This formulation comprised six Chinese herbs: *Rehmannia glutinosa* (Gaertn.) *DC.* (Shu Di Huang), *Cornus officinalis* Siebold & Zucc. (Shan Zhu Yu), *Dioscorea opposita* Thunb. (Shan Yao), *Alisma orientale* (Sam.) Juz. (Ze Xie), *Wolfiporia extensa* (Peck) Ginns (Fu Ling) and *Paeonia suffruticosa* Andrews (Mu Dan Pi). These 6 herbs combined exert "nourishing yin, tonifying kidney, consolidating essence and replenishing qi" to treat kidney-yin deficiency diabetes and chronic metabolic diseases. OS injury drives diabetes and vascular complications, accelerating endothelial dysfunction [175]. LWDHP improves endothelial function and vasodilation in T2DM rats by regulating MDA, protein arginine methyltransferase 1, and Nitric Oxide, inhibiting OS injury to protect the vascular endothelium [176]. Randomized trials show LWDHP combined with Ginkgo Leaf Tablets can effectively relieve T2DM by modulating OS [177]. Additionally, a clinical trial investigating the effect of LWDHP on OS in patients with T2DM demonstrated a significant decrease in plasma levels of carboxymethyllysine and 8-isoprostane, indicating a notable reduction in OS levels [178].

### Yuquan pills (YQP)

Yuquan Pills (YQP) first appeared in Ye Tianshi's *Zhongfutang Selected Effective Prescriptions* (Qing Dynasty), consists of six herbal ingredients: *Pueraria lobata* (Willd.) Ohwi (Ge Gen), *Rehmannia glutinosa* (Gaertn.) DC. (Di Huang), *Ophiopogon japonicus* (Thunb.) Ker Gawl (Mai Dong), *Trichosanthes kirilowii* Maxim. (Tian Hua Fen), *Schisandra chinensis* (Turcz.) Baill. (Wu Wei Zi), and *Glycyrrhiza uralensis* Fisch. (Gan Cao) [179], Sovereign herb *Pueraria lobata* (Willd.) Ohwi (Ge Gen) "raises yang, generates and distributes fluids", directly targeting diabetes with "failure of fluid ascent and dryness-heat damaging fluids". the formula suits qi-yin deficiency with yin-deficiency dryness-heat, especially early-to-middle stage T2DM with prominent "dryness" and "thirst" [180, 181]. The YQP has also accumulated significant experience in the clinical treatment of T2DM. Network pharmacology via PharmMapper indicates YQP modulates PI3K/Akt and MAPK pathways, improving lipid metabolism, OS, and inflammation in T2DM rats, suggesting a potential treatment mechanism [179]. In an HFD/STZ—induced T2DM rat model, YQP (2.16 g/kg) balanced gut microbiota by regulating *Firmicutes*, *Bacteroidetes*, *Ruminococcus*, and *Lactobacillus*, alleviating T2DM related gut dysbiosis [182]. YQP alleviates the symptoms of T2DM by improving glucose and lipid metabolism and reducing inflammation, without any severe adverse events [181]. Notably, YQP has significant potential for treating T2DM and its complications [180].

### Jinqi Jiangtang tablets (JQJT)

Jinqi Jiangtang Tablets (JQJT), formulated based on the therapeutic principles of "clearing heat, invigorating qi, and promoting fluid production" from the *Xiaoke Formula* in the Tang Dynasty classic *Qianjin Fang*, are approved by the National Medical Products Administration (Approval No.: Z10920027) for the treatment T2DM [183]. JQJT comprises *Coptis chinensis* Franch. (Huang Lian), *Astragalus membranaceus* Moench (Huang Qi) and *Lonicera japonica* Thunb. (Jin Yin Hua). These three medicinal herbs are widely used in China and across Asia [184]. *Coptis chinensis* Franch. (Huang Lian clears "dryness-heat" (superficial), *Astragalus membranaceus* Moench (Huang Qi) tonifies "qi deficiency" (root), *Lonicera japonica* Thunb. (Jin Yin Hua) aids heat-clearing. Suitable for diabetes when "dryness-heat remains, qi deficiency is evident". A study on diabetic rats showed that in the experimental renal tissues, JQJT downregulated the expression of Bax, Caspase-3, and cytochrome c while upregulating Bcl-2; by enhancing renal anti-apoptotic activity, this prescription exerts a certain improving effect on the pathological process of T2DM [185]. The key active ingredient palmatine in JQJT can stimulate Fibroblast Growth Factor Receptor 1 phosphorylation, upregulate GLUT-1 expression, promote glucose uptake in IR HepG2 cells, and lower hyperglycaemia in diabetic mice [186]. Experiments have demonstrated that JQJT can upregulate the expression of PPARα in the liver, enhance the metabolism of triglycerides and fatty acids, and ultimately ameliorate T2DM by regulating hepatic glucose and lipid metabolism [187]. Results from randomized controlled trials with 1,425 subjects showed that JQJTT combined with conventional treatment, versus conventional treatment alone or with placebo, has a good effect on regulating glycolipid metabolism and improving IR in T2DM patients [188].

## Potential effects and molecular mechanisms of main components of Chinese herbal medicines on T2DM

Chinese herbal formulas are primarily composed of herbs, whose core lies in their main active ingredients. These active ingredients can function through multiple signaling pathways, including improving insulin sensitivity, protecting β-cell function, reducing inflammatory responses, regulating gut microbiota, and controlling glucose metabolism, thereby alleviating the progression of the disease [189]. This chapter summarizes the active ingredients of traditional Chinese medicines with potential for preventing and treating T2DM over the past five years, highlights the latest advances in their treatment of T2DM, and classifies and analyzes these ingredients (Table 2).

**Table 2:** Main Active Ingredients of Chinese Medicinal Herbs for the Treatment of T2DM 2020 to 2025

No	Natural products	Chemical structure	Types	Dosages	Models	Effects	signal pathway	References
1	Quercetin		In vivo	1.5 g/kg daily for 4 months	HFD/STZ-induced T2DM model	GSH↑, SOD↑, MDA↓, GPX4↓, ROS↓	/	[17]
2	Puerarin		In vivo	80 mg/kg daily for 15 days	HFD/STZ-induced T2DM model	FBG↓, TC↓, TG↓, LDL↓, FINS↑, HDL↑, caspase-3、8、9↓, AIF↓	caspase/AIF/apoptosis	[18]
3	Baicalin		In vivo	25, 50 mg / kg daily for 3 weeks	HFD induced T2DM model	PGC-1α↑, GLUT 4↑, p-p38 MAPK↑, p-AKT↑, p-AS 160↑, GALR 2 ↑	GALR 2/GLUT 4	[204]
4	Ginsenoside Rg1		In vivo	25, 50, 100 mg/kg daily for 8 weeks	HG/HFD/STZ- induces T2DM model	ERK↑, JNKs↓, p-p38↓, Bcl-2↑, Bax↓, Caspase 3↓, Cleaved-Caspase 3↓,TC↓,LDH↓,TG↓, HDL↑	MAPK	[209]
5	Ginsenoside Rb1		In vivo In vitro	20, 50 mg/kg daily for 8 weeks; 20 μM induced 24 h	HFD/STZ- induces T2DM model; Palmitic acid induced IR model in LO2 cells	15-PGDH↓, PGE 2↓, AKT^Ser 473^ ↑, GSK 3β ^Ser 9^↑	15 PGDH/PGE 2/EP 4	[212]
6	Total saponins of momordica charantia	/	In vivo	20, 40, 80 mg/kg daily for 5 weeks	HFD/STZ-induced T2DM model	SOD↑, MDA↓, TG↓, T-CHO↓, LDL-C↓, HDL – C↑, p-AMPK↑、AMPK↑, NF-κB↓	AMPK/NF-κB	[217]
7	Berberine		In vivo In vitro	100, 200 mg/kg daily for 4 weeks; 15, 30 μM induced 24 h	HFD/STZ-induced T2DM model; IR-HepG 2 cell model	GLUT 4↑, IRS-1/PI3K/AKT↑, SOD↑, GPX-px↑, NRF 2↑, NQO 1↑, HO-1↑, ROS↓, MDA↓	AMPK/NRF 2	[16]
8	Total alkaloids of ramulus mori	/	In vivo	400 mg/kg daily for 6 weeks	Obesity model induced by HFD	TG↓, TC↓, TNF-α↓, IL-6↓, PAI-1↓, Ang-1↓, LEP↓, p-ACC↑, p-HSL↑, ATGL↑, PPARα↑, IL-4↑, IL-13↑	/	[226]
9	Astragalus polysaccharides	/	In vivo	700 mg/kg daily for 8 weeks	HFD/STZ-induced T2DM model	GLP↑, STR↑, SGLT-1↓, GLUT 2↓, KCNJ 11↓ T1R2 ↑, Gα gust↑	STR/GLP-1/GLP-1 R	[229]
10	Ganoderma lucidum polysaccharide	/	In vivo	400 mg/kg GLP was suspended in 0.5% CMC-Na solution daily for 4 weeks	HFD/STZ-induced T2DM model	TC↓, TG↓, LDL-C↓, insulin↓, HOMA-IR↓, IL-1β↓, IL-6↓, *Aerococcus*↓, *Ruminococcus*↓, *Corynebactrium*↓, Proteus↓, Blautia↑, *Dehalobacterium*↑, *Parabacteroides*↑, *Bacteroides*↑	/	[237]
11	Pumpkin polysaccharides	/	In vivo	200 mg/kg, daily for 4 weeks	HFD/STZ-induced T2DM model	TC↓, TG↓, LDL-C↓, MDA↓, HDL-C↑, SOD↑, CAT↑	/	[242]
12	Emodin		In vivo In vitro	20, 40, 80 mg/kg daily for 4 weeks; 5, 10, 20 μM induced 6, 12and24h	HFD/STZ-induced T2DM model; Palmitic acid induced IR in L 6 myotube cells	miR-20 b↓, SMAD 7↑	miR-20b/SMAD7 axis	[245]
13	Resveratrol		In vivo	10 mg/kg daily for 6 weeks	HFD/STZ-induced T2DM mode	TXNIP↓, NLRP 3↓, caspase-1↓, IL-1β↓	TXNIP/NLRP 3	[246]
14	Curcumin		In vivo In vitro	100 and 200 mg/kg once daily for eight weeks; 10 μM induced 24 h	Spontaneous hyperglycemia model; Primary mouse islet cell culture	FBG↓, GLP-1↑, *Proteobacteria*↓, *Actinobacteria*↓,*Bacteroidetes*↑, *Firmicutes*↑	/	[255]

### Flavonoids

Flavonoids, including flavones, flavanols, flavanones, and isoflavones, exhibit antioxidant, anti-inflammatory, antiviral, and anticancer effects. They also show potential as T2DM therapeutics by modulating glucose metabolism [190].

#### Quercetin (Que)

Quercetin (Que), a natural pentahydroxyflavone, is found in the roots and rhizomes of *Panax notoginseng* (Burkill) F.H.Chen, *Scutellaria baicalensis* Georgi, and *Ginkgo biloba* L. [5]. The Que in these plants has been shown to have therapeutic effects on diabetes. It improves glucose tolerance and β-cell function, inhibits α-glucosidase and DPP-IV activity, extends GLP-1/GIP half-life, and suppresses the release of IL-1β, IL-4, IL-6, and TNF-α, thereby delaying T2DM progression [191]. In addition, Que protects β-cells from apoptosis by activating Sirt3, regulating ROS and β-cell survival. It increases the protein levels of SOD2, CAT, and Sirt3, reduces the expression of apoptosis markers and the Bax/Bcl-2 ratio, and improves glucose and insulin levels in INS-1 cells and diabetic mice [192]. Que reduces specific bacterial abundance, alters metabolic profiles (increasing L-dopa and S-adenosylmethionine), alleviates IR, repairs the intestinal barrier, and reshapes the gut microbiome in db/db mice [193]. Ferroptosis in pancreatic β-cells contributes to T2DM. Que downregulates GPX4 and induces OS, potentially aiding T2DM treatment [17].

#### Puerarin (Pue)

Puerarin (Pue) is an active isoflavone glycoside compound extracted from the dried roots of *Pueraria montana* var. lobata (Willd.) Maesen & S.M.Almeida ex Sanjappa & Predeep or *Pueraria thomsonii* Benth., both belonging to the Fabaceae family [194]. Its efficacy in "promoting the production of body fluids and relieving thirst," especially for symptoms like excessive thirst and dry mouth, has been documented and recognized in traditional herbal texts, including the *Shennong Bencao Jing*. Modern pharmacological studies indicate that Pue lowers blood glucose by protecting β-cells, improving IR, inhibiting α-glucosidase, and combating OS and inflammation, enhancing therapeutic efficacy [195]. Pue regulates glucose and lipid metabolism disorders and IR. It activates AMPK and the PI3K/Akt pathway in the liver, and increases GLUT4 mRNA expression [196]. Liu et al. [197] gave 300 mg/kg/d of Pue for 4 weeks to a HPD/STZ -induced T2DM rat model, significantly reducing FBG, HbA1c, and TG levels. Pue lowers FBG, TC, TG, and LDL, while increasing FINS (Fasting blood Insulin) and HDL, and inhibits pancreatic β-cell pyroptosis [18]. In addition, Pue reduces FBG, IR, and FINS in T2DM by downregulating adipose differentiation-related protein mRNA in adipose tissue, showing potential for improving IR and blood glucose control [198].

#### Baicalein (Bai)

Baicalin (Bai) is a major active flavonoid glycoside compound extracted from *Scutellaria baicalensis* Georgi, a plant of the Lamiaceae family, specifically from its dried roots [199]. The traditional herbal text *Mingyi Bielu* explicitly records that Scutellaria baicalensis has the effect of clearing heat and purging fire, especially for clearing heat in the lung and stomach, and can be used to treat diabetes [200]. In recent years, it has been found that Bai has hypoglycaemic, lipid-lowering, and metabolism-improving effects [201]. Yan et al. [202] found that Bai improved hyperglycemia, glucose tolerance, and reduced liver and bile bile acid levels in diabetic mice. It also inhibited cholesterol 7α-hydroxylase and FXR overexpression, reducing lipid accumulation and enhancing glucose and lipid metabolism regulation. Additionally, Bai (25 mg/kg/d and 50 mg/kg/d) and GALR2 antagonist M871 (10 mg/kg/d) improved metabolic function in obese mice by reducing hyperglycemia and enhancing insulin sensitivity. The treatment downregulated PGC-1α while activating insulin signaling (p-p38MAPK/p-AKT/p-AS160) and GLUT4 translocation, suggesting therapeutic potential for metabolic disorders [203]. In vitro experiments showed that Bai can promote cellular glucose uptake, enhance PGC1α-GLUT4 axis activity, and activate the p38 MAPK and AKT pathways, and through the GALR2-GLUT4 pathway, it can achieve the preventive effect on IR [204].

### Saponins

Saponins, composed of sapogenins and sugars/organic acids, are classified as triterpenoid and steroidal saponins [205]. TCM saponins exhibit multi-target pharmacology with minimal side effects, offering potential for preventing and treating T2DM through diverse mechanisms.

#### Ginsenoside Rg1 (GRg1)

Ginsenoside Rg1 (GRg1) is a major active saponin compound extracted from *Panax ginseng* C.A.Mey. The Compendium of Materia Medica records that ginseng has the effect of promoting the production of body fluids and nourishing blood, and it is used to alleviate the thirst associated with "Xiao ke". It exhibits anti-apoptotic, anti-inflammatory, antioxidant, and antidepressant properties. Research indicates that GRg1 can alleviate inflammation and OS in T2DM rats and improve IR [206]. In T2DM rats, GRg1 can reduce blood glucose, improve IR, and regulate lipid metabolism indicators (including TCHO, TG, LDL-C), while exhibiting hepatic protection and improving T2DM-related pathological indicators [207]. Experimental evidence from in vitro investigations has established that chronic insulin treatment of differentiated C2C12 muscle cells induces IR, resulting in reduced glucose uptake. However, GRg1 significantly improved glucose uptake in these muscle cells and increased GLUT4 abundance by activating the AMPK pathway, which is crucial for T2DM prevention [208]. Su et al. [209] found GRg1 dose-dependently enhanced ERK phosphorylation, suppressed JNKs/p38 phosphorylation, increased Bcl-2, and decreased Bax/Caspase-3 in T2DM rat brains, indicating neuroprotection via MAPK signaling and apoptosis modulation.

#### Ginsenoside Rb1 (GRb1)

Ginsenoside Rb1 (GRb1) and GRg1 share the same source and traditional applications in treating diseases, and they account for approximately 20% of the total saponins in *Panax ginseng* C.A.Mey. [210]. GRb1 improves hepatic glucose metabolism, IR, and hepatic steatosis in T2DM mice by regulating signal transducer and activator of transcription 3 signaling, thereby reversing the abnormal expression of glycolytic and gluconeogenic enzymes [211]. In vitro, it enhances hepatic glycogen synthesis in T2DM mice by increasing AKT and GSK3β phosphorylation. Molecular docking shows high affinity for 15-hydroxyprostaglandin dehydrogenase (15-PGDH), suggesting a 15-PGDH—dependent glycogen—promoting pathway [212]. Moreover, GRb1 alleviates T2DM by reducing blood glucose, IR. It modulates gut microbiota and faecal metabolites, cutting harmful fatty acids. It may function as a prebiotic, targeting diabetes and metabolism related gut microbes and metabolites [213].

#### Total saponins of Momordica charantia (TSMC)

Total Saponins of Momordica charantia (TSMC), extracted from the plant *Momordica charantia* L., are known for their traditional uses in clearing heat, detoxifying, and quenching thirst, as recorded in the Compendium of Materia Medica. They are particularly effective for alleviating irritability and have been used to treat diabetes (referred to as "Xiao ke syndrome" in TCM [214]. The total saponins extracted from TSMC are key active components. Studies show that TSMC significantly impacts lipid metabolism, OS, and the insulin signaling pathway in T2DM rats. TSMC derived total saponins have anti-inflammatory effects, inhibiting pro-inflammatory factors (COX-2, IL-1β, IL-6) and activating PPAR-γ, showing promise for diabetes management [215]. In animals, TSMC treatment increases SOD and catalase in the liver and pancreas, reduces MDA and p-IRS-1, and improves glucose related parameters [216]. Wang et al. [217] reported that 400 mg/kg TSMC in T2DM mice restores body weight, lowers FBG, improves IR and raises liver p-AMPK, likely through AMPK/NF-κB-signalling mediated energy metabolism regulation.

### Alkaloids

Alkaloids are a class of nitrogen-containing secondary metabolites widely distributed in various plants and certain organisms. Pharmacologically, they exhibit anti-inflammatory activities, hypoglycaemic effects, and lipid-modulating properties; these multi-target bioactivities collectively endow alkaloids with considerable potential for the treatment of T2DM.

#### Berberine (BBR)

Berberine (BBR) is a natural alkaloid found in plants like Berberis and *Coptis chinensis* Franch. (Huang Lian), especially in their roots and bark. It has been traditionally used in medicine to manage diabetes and its symptoms (excessive thirst, urination, and appetite) and to address related “damp-heat” imbalances [218]. Modern pharmacological studies have shown that it possesses various activities such as anti-diabetic, lipid-lowering, antibacterial, anti-inflammatory, and anti-cancer properties [219]. Guo et al. [220] evaluated indicators including HbA1c, fasting plasma glucose, fasting insulin, HOMA-IR, TC, LDL, IL-6, and TNF-α. Their findings demonstrate that BBR improves IR, regulates lipid metabolism, and suppresses pro-inflammatory factors, supporting its role in T2DM treatment. This study (50 studies, 4,150 subjects) showed BBR alone reduces Fasting Plasma Glucose, 2-Hour Postprandial Blood Glucose, LDL-C, TC and TG; combined with hypoglycemic drugs, it improves Fasting Plasma Glucose, 2-Hour Postprandial Blood Glucose, HbA1c and other metabolic markers. Commonly used at 0.9 g/d -1.5 g/d for 1–3 months, BBR (alone or combined) has great potential in T2DM treatment; future studies should expand scope and explore its mechanism with hypoglycemic drugs to optimize strategies [221]. In addition, BBR acts in the intestine via the Phospholipase Cγ2 signaling pathway to induce GLP-1-mediated insulin secretion; meanwhile, it exerts a certain protective effect against mitochondrial damage, reduces adipose tissue fat content, and decreases body OS level. These effects may contribute to improving disease-related pathological conditions [222].

#### Total alkaloids of Ramulus Mori (TARM)

Total Alkaloids of Ramulus Mori (TARM), extracted from the dried young branches of *Morus alba* L., are traditionally used to treat diabetes (Xiao ke) with symptoms like excessive thirst, frequent urination, fatigue, and weight loss, as recorded in the Compendium of Materia Medica [223]. Its polyhydroxyalkaloids, key glycosidase inhibitors, reduce/prevent gut microbiota imbalances from glucose-lipid metabolism disorders, inflammation and OS, effectively lowering blood glucose and alleviating hyperglycemia-induced organ damage. TARM was approved for improving T2DM in 2020 [224]. The active ingredients can exhibit hypoglycaemic activity by regulating glucose, amino acid, and lipid metabolism, and almost no adverse reactions [225]. Sun et al. [226] administered TARM at 400 mg/kg to T2DM mice reduced body weight and serum lipid levels (TG and TC), downregulated inflammatory factors (IL-6, TNF-α, MCP-1, F4/80) in adipose tissue, and upregulated anti-inflammatory cytokines (IL-4, IL-10, IL-13), highlighting its potential in regulating lipid metabolism and alleviating adipose inflammation. Additionally, combining TARM with metformin significantly improved blood glucose control in T2DM patients, reducing HbA1c, FBG, average blood glucose, and glucose variability [227].

### Polysaccharides

Chinese medicinal polysaccharides, with characterized by low toxicity, high safety, and multi-target mechanisms, exhibit a spectrum of biological activities including anti-inflammatory, antioxidant, anti-fibrotic, immunomodulatory, and anti-tumor effects. These versatile bioactivities enable them to play a pivotal role in the management of T2DM.

#### Astragalus polysaccharides (APs)

Astragalus polysaccharide (APs) is the main active ingredient extracted from the stems or dried roots of Astragalus membranaceus. It exerts antioxidant, antihypertensive, and antitumor activities through mechanisms such as vascular regulation, immune enhancement, and pro-apoptotic effects [228]. In the intestines of T2DM, APs can enhance the signaling of GLP-1 and sweet taste receptors, while reducing the expression of SGLT-1 and GLUT2 [226]. APs increased GLP-1 and sweet receptor signaling in the intestines of T2DM rats while reducing SGLT-1 and GLUT2 glucose transporter expression. This promotes insulin secretion and alleviates T2DM symptoms through the intestinal glucose transporter and STRs/GLP-1 pathway [229]. Wei et al. [230] found APs can reduce IR in T2DM. It upregulates miR-203a—3p, decreases GRP78, and modulates the Endoplasmic Reticulum Stress pathway. In vivo, APs lowers blood glucose and insulin, improves glucose intolerance and IR, regulates lipids, and reduces OS [231]. In vitro, APs inhibits *Shigella*, promotes beneficial *Cocci* and *Lactobacillus*, repairs intestinal barrier, and improves metabolism, reducing inflammation, OS, and organ damage in T2DM mice [232].

#### Ganoderma lucidum polysaccharides (GLPs)

Ganoderma lucidum (GLPs), also known as "Lingzhi" in China, has been a health-promoting herbal food (with both medicinal and edible properties) and a traditional medicinal material in Southeast Asian countries for centuries. In TCM and Japanese medicine, it is used to treat diseases such as hypertension, hepatitis, chronic bronchitis, and cancer [233]. GLPs has been shown to have therapeutic benefits in diabetes [234]. GLPs significantly lowers blood glucose, promotes insulin secretion, improves glucose tolerance, and regulates blood lipids. Studies show GLPs increases calmodulin (CaM), heat shock transcription factor 1 (HSF1), family with sequence similarity 3 member C (FAM3C), and p-AKT/AKT expression in T2DM mouse hepatocytes, indicating it modulates diabetic lipid metabolism via the FAM3C-HSF1-CaM pathway [235]. Xue et al. [236] administered GLPs (200 mg/kg, 400 mg/kg, and 800 mg/kg) to T2DM rats showed high-dose GLPs most effectively reduced blood glucose and activated antioxidant enzymes (GSH-PX, CAT, SOD), improving hemodynamic and antioxidant activity in heart tissue. In vitro experiments demonstrated that GLPs can reverse the metabolic disorders of amino acids, inflammatory mediators, carbohydrates, and nucleic acids in the gut microbiota of T2DM rats [237].

#### Pumpkin polysaccharides (PPs)

Pumpkin polysaccharides (PPs) are brown powdery solids, serving as effective components with unique biological activities derived from pumpkin plants [238]. PPs are heteroglycans composed of glucose, galactose, rhamnose, arabinose, xylose, and glucuronic acid monomers [239]. PPs and Pue synergistically enhance glucose tolerance and reduce IR in T2DM mice. They regulate lipid levels (lowering TG, TC, LDL; increasing HDL) and mitigate OS (reducing ROS, MDA; increasing GSH, SOD), with Nrf2 and PI3K pathways involved [240]. Research shows that administering PPs to KKAY mice on a HFD effectively reduces weight gain and lowers plasma insulin, TG, LDL-C, and blood glucose levels while increasing HDL-C and liver glycogen. These findings suggest that PPs have significant lipid-lowering and blood-glucose-lowering effects [241]. Furthermore, during the pathological progression of T2DM, PPs can downregulate the levels of abnormally elevated indicators such as blood glucose, insulin, total blood lipids, and MDA, upregulate the level of HDL-C—a protective lipid indicator—and enhance the antioxidant activities of SOD and CAT [242].

### Others

#### Emodin (Emo)

Emodin (Emo), an important anthraquinone compound isolated from the roots and rhizomes of Rheum plants (Polygonaceae), is also found in plants such as Polygonum cuspidatum rhizomes and *Cassia obtusifolia* L. It exhibits significant efficacy against key syndrome types in the treatment of “Xiao ke”, including "excessive stomach heat", "intestinal dryness with fluid injury", and "blood stasis obstruction". Emo possesses anti-inflammatory and immunomodulatory effects, and simultaneously exhibits a certain degree of activity in blood glucose regulation [243]. Emo demonstrates limited systemic bioavailability, with a reported value of 3.2%. The majority of the administered dose (56%) is excreted unchanged in feces. The absorbed fraction undergoes extensive first-pass metabolism, forming hydroxylated and glucuronidated derivatives that show preferential distribution to the kidneys. Hydroxylated metabolites are eliminated through both urinary and fecal routes, while glucuronidated conjugates are excreted primarily via renal clearance [244]. In addition, Xiao et al. [245] measured FBG and lipid levels in T2DM before and after gastric administration of Emo and found that Emo effectively improves glucose metabolism and counters IR.

#### Resveratrol (Res)

Resveratrol (Res), a major stilbenoid polyphenol extracted from the rhizomes of *Persicaria acuminata* (Kunth) M.Gómez, has explicit application records in TCM. TCM classics document that *Polygonum cuspidatum* can regulate "Xiao ke" and symptoms like "fan ke" (polydipsia), with direct references in *Diannan Materia Medica*. Its effects of "clearing heat-toxin, removing dampness for jaundice, and activating blood to resolve stasis" are used to improve the "heat-stasis-dampness" pathological state in “Xiao ke”. It can serve as one of the potential candidate drugs for the treatment of T2DM [246, 247]. Res helps regulate T2DM blood glucose levels by increasing the expression of *Slc2a4*/GLUT4 in muscle and *Slc2a2*/GLUT2 in the liver [248]. IR impairs glucose control in T2DM by reducing uptake and increasing liver output. GLUT4 (*Slc2a4*) and GLUT2 (*Slc2a2*) are key for muscle and liver glucose flux. Res improves glucose homeostasis, enhances GLUT4, GLUT2, and SIRT1, and regulates liver output. It also increases *Slc2a4*/GLUT4 in muscle and *Slc2a2*/GLUT2 in the liver, aiding blood glucose regulation [249]. This meta-analysis included 25 articles involving a total of 1,171 participants. The results showed that Res significantly reduced glycosylated hemoglobin, TC, and LDL-C, and also had a significant effect on HDL-C. The conclusion indicates that Res plays a significant role in regulating lipid and glucose metabolism. Future large-scale, well-designed trials are needed to further validate these results, providing more references for clinicians in the use of Res [250].

#### Curcumin (Cur)

Curcumin, a major active polyphenolic compound extracted from the rhizomes of *Curcuma longa* L. (Zingiberaceae), has the efficacy of "activating blood circulation and promoting qi flow" in TCM theory, and is often used to improve the blood stasis syndrome accompanied by the late stage of "Xiao ke". Numerous studies have demonstrated that it possesses anti-inflammatory, antioxidant, anti-atherosclerotic, and anti-diabetic properties. [251]. Experiments have demonstrated that Cur can reduce the levels of HbA1c, MCP-1 and TNF-α in diabetic rats and inhibit the NF-κB pathway [252]. Cur can significantly suppress the production of TGF-β1 and type II TGF-β receptor, and block the AMPK/P38 MAPK pathway [253]. Ultimately achieving therapeutic effects. Cur reduces MDA and increases SOD levels in alloxan-treated islet cells, demonstrating its potential for islet cell protection and T2DM treatment [254]. Experiments have demonstrated that tetrahydrocurcumin can upregulate the expression of GLP-1, reduce the relative abundance of *Actinobacteria* and *Proteobacteria*, and normalize the *Firmicutes/Bacteroidetes* ratio. These regulatory effects collectively promote insulin secretion and exert a hypoglycemic effect, thereby contributing to blood glucose control [255].

## Toxicology and side effects

The global T2DM patient population is large and growing, with high complication risks. Evaluating T2DM drug safety and efficacy is vital. In TCM, conventional doses of herbs are generally considered safe, but toxicity research on their prescriptions and active ingredients is notably insufficient, relevant information in package inserts needs refinement, and existing studies are mostly limited to acute and conventional toxicity. The U.S. Food and Drug Administration demands new T2DM drugs to not possess unacceptable ischemic cardiovascular risks [256]. This reflects high level of concern regarding the potential toxic side effects that glucose-lowering medications may cause.

The international community remains highly concerned about TCM safety. Most T2DM treatment trials report no significant adverse reactions. However, Zhang Q.J. et al. [162] reported 4 cases of loose stools in the DCHD combination group, 5 of abdominal distension and 2 of nausea in the conventional group; gastrointestinal adverse reactions all resolved spontaneously with no serious events, indicating DCHD is relatively safe when used properly. Moreover, hypoglycemia is among the most common and serious adverse events. Wang et al. [257] found no severe hypoglycemia in Phases I and II trials exploring dosage-efficacy relationships of GQD in T2DM. Additionally, 15 randomized controlled trials on SQJTG showed no liver or kidney damage, indicating it significantly reduces hypoglycemia risk and has efficacy and safety as T2DM adjuvant therapy [171]. Thus, during clinical administration, it is crucial to closely monitor whether the use of drugs has potential side effects.

In active monomer research, considering their potential toxicological properties and side effects is crucial. Reports on Que's carcinogenicity in mammals and genotoxicity to Salmonella show that typical doses are unlikely to cause adverse reactions, with its human application safety recognized [258]. In a trial, 36 healthy subjects took 200 mg, 400 mg, or 600 mg of Bai tablets. Given once daily on days 1 and 10, and three times daily from days 4–9, all adverse events were mild and self—resolving, showing good safety [259]. In a study of 84 new T2DM patients on BBR, 20 had transient gastrointestinal side effects like nausea and diarrhoea. Short term use may not be serious, but long term may harm gut microbiota, potentially leading to gastrointestinal dysfunction [260]. A randomized trial showed BBR, though effective for lowering blood sugar, increases hypoglycaemia risk when combined with oral hypoglycaemics [261]. Three randomized controlled trials with 50 T2DM participants, using different oral resveratrol doses vs placebo, showed no adverse events, indicating good tolerability and potential safety of 10 mg/d to1000 mg/d for 4 weeks to 5 weeks. However, evidence is insufficient to support resveratrol supplements for treating T2DM in adults [262].

Current evidence shows herbal medicines and their active ingredients are relatively safe, but limited data prevents accurate conclusions. Thus, well-designed quantitative studies with clinical evidence are urgently needed to systematically assess potential toxicity and ensure clinical medication safety.

## Conclusions and perspectives

T2DM has emerged as the most prevalent life-threatening chronic disorder worldwide. The disease's multifactorial pathogenesis necessitates personalized therapeutic approaches. Persistent hyperglycaemia induces progressive multi-organ dysfunction and predisposes patients to severe complications [263]. First-line oral hypoglycemic agents, including biguanides and sulfonylureas, are limited by non-specific biodistribution, short plasma half-lives, and significant adverse effects that compromise treatment efficacy and patient compliance [264–267]. T2DM pathogenesis involves inflammation, OS, and autophagy, highlighting the need for new drugs. TCM can relieve T2DM symptoms, but challenges limit the clinical use of TCM compounds in patients.

Firstly, current drugs for T2DM primarily exert their effects by enhancing insulin secretion, protecting pancreatic β-cells, and regulating metabolic processes. For example, metformin—one of the first-line medications for T2DM—improves insulin sensitivity, yet its antioxidant activity remains limited. In contrast, TCM acts via distinct and complementary mechanisms. Notably, chronic inflammation IL-6 and TNF-α is a key driver of T2DM progression, and TCM can modulate this inflammatory response through pathways such as NF-κB, TLR4/NF-κB, and MAPK [268, 269]. Moreover, TCM better modulates OS by targeting both Nrf2 and NF-κB pathways [270]. Antioxidant drugs have limited impact on Ferroptosis, linked to lipid peroxidation; On the other hand, TCM manages iron, has antioxidant effects, and inhibits ferroptosis [271, 272]. Pyroptosis relates to inflammation and SGLT-2 inhibitors reduce inflammation and indirectly suppress pyroptosis, while TCM directly inhibits the NLRP3 inflammasome to do so [273]. Another issue that worsens T2DM is autophagy. The drug Rapamycin is used for autophagy but has side-effects, whereas, TCM promotes autophagy via AMPK, mTOR, and PI3K/Akt to protect β-cells [274, 275]. Gut microbiota imbalance also causes IR. Probiotics/prebiotics have safety concerns, while TCM modulates microbiota and intestinal function [276]. Overall, TCM, with its multi—faceted actions, comprehensively regulates key T2DM related processes. The Jianyutangkang—metformin combo, for example, addresses both blood sugar and lipid problems, compensating for drug drawbacks [277]. Future work should explore TCM—drug synergies, optimize treatments, and clarify drug limitations for better patient care.

Secondly, as an alternative for T2DM, TCM faces certain limitations in its dynamic processes of absorption, distribution, metabolism, and excretion. Que, a hydrophobic compound, has low solubility in water, gastric, and intestinal fluids. Its poor solubility, stability, and absorption lead to low bioavailability (about 10%) [278]. However, when encapsulated in a Tween 80-stabilized oil in water nanoemulsion and given orally to rats, it reduces blood glucose, OS and inflammation, showing potential as a treatment for T2DM [279]. Chronic toxicity tests show that high—dose Que (over 400 mg/kg daily for 410 days) causes no significant organ changes in rats, indicating low toxicity [280]. BBR has poor solubility and permeability. P-glycoprotein efflux and metabolism limit its bioavailability to < 1%, hampering clinical use [281]. A new food—grade delivery system tested in 10 volunteers significantly boosted AUC0—24 and Cmax (p < 0.05), with six—fold higher bioavailability, showing it to be highly promising [282]. In rat tests by Jia et al.[283] found that in rats, the model group had increased BBR C_max_, t_1/2_, AUC _(0—t)_ and reduced oral clearance when given 20 mg/kg orally, suggesting T2DM patients may need dosage adjustment. Ginsenoside monomers suffer low bioavailability (< 5%) from gastric degradation, overcome by novel liposomal formulation (soybean phosphatidylcholine-based) achieving 11.8-fold enhancement [284]. The complexity of TCM's chemical components makes inferring bioavailability difficult. Most studies are at the animal and cellular levels, lacking real-world data and randomized trials to validate their efficacy and safety in treating T2DM. Potential toxic side effects and small-scale, short-duration experiments hinder comprehensive risk assessment. Translating findings into clinical practice remains challenging. Future large-scale trials are essential to systematically evaluate safety, efficacy, and bioavailability improvement methods, ensuring a reliable basis for clinical use.

Thirdly, the safety and standardization of TCM are controversial. Herbal medicines come from diverse raw materials. Differences in origin, harvesting season, and processing can cause composition and efficacy variations. For instance, the active components in *Salvia miltiorrhiza* Bunge (Dan shen) differ by origin [285]. Herbal medicine lacks standardized production protocols, causing inconsistent quality. Example: Silymarin extraction ranges from undetectable (25% ethanol) to optimal (70% ethanol) [286]. In future studies, it will be necessary to use multi-omics technology to analyse the correlation rules among "process-ingredients-efficacy", so as to provide a theoretical basis for the production process.

Fourthly, although this study explores the molecular mechanisms underlying the regulation of T2DM by TCM, the integration of relevant signaling pathways remains to be improved—current research mostly stays at the preliminary verification stage, lacking systematic mechanistic analysis. The verification of TCM’s downstream effector mechanisms in improving T2DM is still at the "phenomenon description level": in studies related to gut microbiota- IR, only the changing trends of microbiota abundance can be observed, and the causal relationship between them has not been clarified; in research on ferroptosis-pancreatic β-cell apoptosis, only the fluctuation characteristics of iron metabolism have been recorded, with insufficient in-depth exploration of core regulatory molecules. Furthermore, the verification of the efficacy and safety of the TCM compounds and active ingredients included in this study mostly relies on in vitro experimental and animal model data—only a small portion have undergone systematic evaluation through large-scale, multi-center randomized controlled clinical trials, or obtained official recognition from authoritative institutions regarding their therapeutic potential for T2DM. This research status leads to a significant gap between existing conclusions and actual clinical needs, making it difficult to directly provide high-quality evidence-based support for clinical treatment. Therefore, it is urgent to further advance clinical translation research to bridge the gap between basic research and clinical application.

Overall, this article comprehensively reviews recent research regarding the molecular mechanisms underlying the treatment of T2DM by TCM. It points out that although the AMPK pathway has been extensively investigated, other pathways still require further exploration [287]. From aspects like regulating IR, restoring pancreatic β—cell function, and improving glucose and lipid metabolism, it details TCM research progress in inflammation, OS, ferroptosis, pyroptosis, autophagy, and gut microbiota, and summarizes TCM's advantages as a medicinal and edible option for T2DM treatment. In the future, efforts should be made to strengthen multidisciplinary collaboration, conduct high quality clinical studies, establish standardized production systems, and improve quality control to enhance TCM's role in global healthcare. This will drive traditional medicine innovation and offer new ideas for modern medicine.

## Data Availability

No datasets were generated or analysed during the current study.

## Abbreviations

ACSL 4: Acyl-CoA synthetase long-chain family member 4
AGEs: Advanced glycation end products
ATG7: Autophagy related gene 7
AA: Arachidonic acid
AdA: Adrenergic acid
APS: Astragalus polysaccharide
BA: Bile acids
BCAA: Branched-chain amino acid
BBR: Berberine
Bai: Baicalein
CAT: Catalase
Bcl-2: B-cell lymphoma-2
BMI: Body mass index
Cur: Curcumin
DCHD: Dachaihu decoction
FBG: Fasting blood glucose
FINS: Fasting blood insulin
FXR: Farnesoid X Receptor
FFA: Free fatty acids
FoxO 1: Forkhead box protein O1
GQD: Gegen Qinlian decoction
GRg1: Ginsenoside Rg1
GRb1: Ginsenoside Rb1
GSH: Glutathione
GLUT4: Glucose transporter 4
HbA1c: Haemoglobin A1C
HDL-C: High-density lipoprotein cholesterol
HFD/STZ: High-fat diet/streptozotocin
HLJDD: Huanglian Jiedu decoction
HOMA-IR: Homeostasis model assessment-insulin resistance
IR: Insulin resistance
InsR: Insulin receptor
IRS: Insulin receptor substrate
IAPP: Islet amyloid polypeptide
JLDG: Jinlida granules
JQJT: Jinqi Jiangtang Tablets
LPS: Lipopolysaccharide
LDL-C: Low-density lipoprotein cholesterol
LWDHP: Liuwei Dihuang pills
MCP-1: Monocyte chemoattractant protein-1
mTORC: Mammalian target of rapamycin complex
MDA: Malondialdehyde
MMP: Matrix metalloproteinases
NaAsO_2_: Sodium arsenite
OS: Oxidative stress
PUFA: Polyunsaturated fatty acids
PE: Phosphatidylethanolamine
POMC: Proopiomelanocortin
PGC-1α: Peroxisome proliferator-activated receptor gamma coactivator 1 alpha
PYY: Peptide YY
Pue: Puerarin
15-PGDH: 15-Hydroxyprostaglandin dehydrogenase
PDK1: 3-phosphoinositide-dependent protein kinase 1
PIP3: Phosphatidylinositol-3,4,5-Trisphosphate
Que: Quercetin
ROS: Reactive oxygen species
Res: Resveratrol
Rab: Ras-related in brain GTPases inactivation
SOD: Superoxide dismutase
SCFAs: Short-chain fatty acids
SQJTG: Shenqi Jiangtang granules
SNAREs: Soluble N-ethylmaleimide-sensitive factor attachment protein receptors
SIRT1: Sirtuin 1
TARM: Total alkaloids of Ramulus Mori
T2DM: Type 2 diabetes mellitus
TCM: Traditional Chinese medicine
TC: Total cholesterol
TG: Triglyceride
TSMC: Total saponins of Momordica charantia
YYD: Yuye decoction
YQP: Yuquan pills
ZYD: Zengye decoction
